# Targeted sequencing of *Enterobacterales* bacteria using CRISPR-Cas9 enrichment and Oxford Nanopore Technologies

**DOI:** 10.1128/msystems.01413-24

**Published:** 2025-01-08

**Authors:** Hugh Cottingham, Louise M. Judd, Jessica A. Wisniewski, Ryan R. Wick, Thomas D. Stanton, Ben Vezina, Nenad Macesic, Anton Y. Peleg, Iruka N. Okeke, Kathryn E. Holt, Jane Hawkey

**Affiliations:** 1 Department of Infectious Diseases, School of Translational Medicine, Monash University, Melbourne, Victoria, Australia; 2 Centre to Impact AMR, Monash University 2541 , Melbourne, Victoria, Australia; 3 Infection Program, Monash Biomedicine Discovery Institute, Department of Microbiology, Monash University, Melbourne, Victoria, Australia; 4 Department of Pharmaceutical Microbiology, Faculty of Pharmacy, University of Ibadan, Ibadan, Nigeria; 5 Department Infection Biology, London School of Hygiene & Tropical Medicine, London, United Kingdom; The University of Hong Kong, Sai Ying Pun, Hong Kong, China

**Keywords:** Oxford Nanopore, CRISPR-Cas9 enrichment, *Klebsiella*, *Enterobacterales*, metagenomics

## Abstract

Sequencing DNA directly from patient samples enables faster pathogen characterization compared to traditional culture-based approaches, but often yields insufficient sequence data for effective downstream analysis. CRISPR-Cas9 enrichment is designed to improve the yield of low abundance sequences but has not been thoroughly explored with Oxford Nanopore Technologies (ONT) for use in clinical bacterial epidemiology. We designed CRISPR-Cas9 guide RNAs to enrich the human pathogen *Klebsiella pneumoniae*, by targeting multi-locus sequence type (MLST) and transfer RNA (tRNA) genes, as well as common antimicrobial resistance (AMR) genes and the resistance-associated integron gene *intI1*. We validated enrichment performance in 20 *K*. *pneumoniae* isolates, finding that guides generated successful enrichment across all conserved sites except for one AMR gene in two isolates. Enrichment of MLST genes led to a correct allele call in all seven loci for 8 out of 10 isolates that had depth of 30× or more in these regions. We then compared enriched and unenriched sequencing of three human fecal samples spiked with *K. pneumoniae* at varying abundance. Enriched sequencing generated 56× and 11.3× the number of AMR and MLST reads, respectively, compared to unenriched sequencing, and required approximately one-third of the computational storage space. Targeting the *intI1* gene often led to detection of 10–20 proximal resistance genes due to the long reads produced by ONT sequencing. We demonstrated that CRISPR-Cas9 enrichment combined with ONT sequencing enabled improved genomic characterization outcomes over unenriched sequencing of patient samples. This method could be used to inform infection control strategies by identifying patients colonized with high-risk strains.

**IMPORTANCE:**

Understanding bacteria in complex samples can be challenging due to their low abundance, which often results in insufficient data for analysis. To improve the detection of harmful bacteria, we implemented a technique aimed at increasing the amount of data from target pathogens when combined with modern DNA sequencing technologies. Our technique uses CRISPR-Cas9 to target specific gene sequences in the bacterial pathogen *Klebsiella pneumoniae* and improve recovery from human stool samples. We found our enrichment method to significantly outperform traditional methods, generating far more data originating from our target genes. Additionally, we developed new computational techniques to further enhance the analysis, providing a thorough method for characterizing pathogens from complex biological samples.

## INTRODUCTION

Effective and rapid characterization of antimicrobial-resistant bacterial pathogens is crucial for improving patient outcomes and containing outbreaks in hospital settings. Current gold-standard characterization methods, such as whole-genome sequencing, MALDI-TOF mass spectrometry, and antimicrobial susceptibility testing, rely on time-consuming bacterial culture (1). Modern high-throughput sequencing technologies, such as Illumina and Oxford Nanopore Technologies (ONT), have the capacity to vastly improve characterization speed by bypassing bacterial culture and sequencing pathogenic DNA directly from patient samples. However, many sample types, including fecal, saliva, nasal, and vaginal specimens, contain pathogen DNA at <10% abundance of total DNA (2–4). This often leads to insufficient pathogen sequence data for effective characterization. Several methods have been developed to enrich low-abundance pathogen DNA prior to sequencing, but all have significant limitations. Host DNA depletion methods (e.g., saponin enrichment and CpG methylated DNA removal) are not selective for specific bacteria, and those based on differential cell lysis require fresh, unfrozen samples to be effective (5–8). Amplicon sequencing methods, such as selective whole-genome amplification, can take days to complete and often cannot target multiple different pathogens at once (9, 10). Hybrid capture-based methods suffer from high financial costs and a lack of target flexibility (11, 12). ONT’s adaptive sampling shows promise for depleting unwanted DNA during sequencing, but has thus far been unable to substantially increase absolute numbers of target sequences and often leads to premature flowcell degradation (13–17).

**Fig 1:**
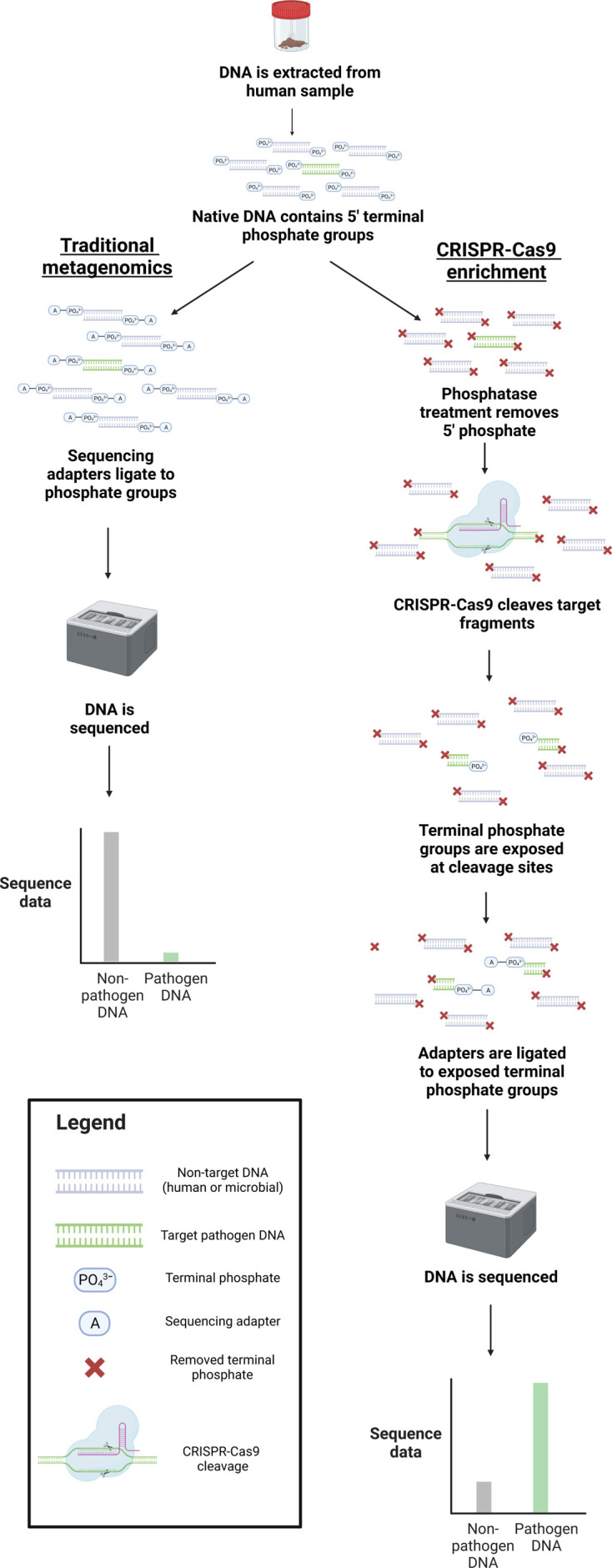
Library preparation differences between unenriched and CRISPR-Cas9 enriched sequencing. During unenriched sequencing, sequencing adapters are ligated to native terminal phosphate groups on DNA molecules to allow for sequencing. During CRISPR-Cas9 enrichment, native phosphate groups are removed from all DNA molecules so that adapters cannot ligate. CRISPR-Cas9 is then used to cleave molecules of interest, exposing their terminal phosphate groups and allowing for specific adapter ligation and sequencing.

CRISPR-Cas9 enrichment allows the selective sequencing of DNA fragments containing a chosen 23 bp target sequence. This approach is applied immediately following DNA extraction and begins with the removal of terminal phosphate groups from genomic DNA, preventing phosphate-dependent sequencing adapters from ligating to DNA molecules so they will not be available for sequencing (Fig. 1). A pool of CRISPR-Cas9 guide RNAs (guides) is then used to direct Cas9 cleavage of sequences with a complementary sequence, exposing internal phosphate groups so that sequencing adapters can then selectively ligate to cleaved molecules, making them available for sequencing (Fig. 1).

The first published use of CRISPR-Cas9 for enrichment of bacterial DNA was employed by Quan et al., who targeted 127 AMR genes in *Staphylococcus aureus* and *Enterococcus faecium* (18). While this study demonstrated effective detection of low-abundance pathogens, it also highlighted the limitations of using short-read sequencing. Illumina short-read sequencing requires adapters to be present at both ends of the molecule, therefore requiring a minimum of two guide RNAs to cleave at nearby sites to create a molecule with adapters at both ends. Short-read lengths also generate minimal information on the genetic context of the target genes (18). ONT platforms allow for theoretically unlimited read lengths, which could vastly improve the amount of genetic information obtained from each enrichment site. Previous bacterial studies combining CRISPR-Cas9 enrichment with long-read sequencing showcased these benefits, but primarily focused on AMR genes (19–21).

Here, we chose to focus on the enrichment of *Klebsiella pneumoniae* and closely related species comprising the *K. pneumoniae* species complex (KpSC) (22, 23), which are associated with high levels of AMR and virulence that can lead to severe cases of sepsis, pneumonia, and urinary tract infections (24). Additionally, this species belongs to the order *Enterobacterales*, which accounts for a large proportion of carbapenem-resistant infections in hospitals (25). We present an implementation of CRISPR-Cas9 enrichment and ONT sequencing targeting transfer RNA (tRNA), AMR, and multi-locus sequence type (MLST) genes in *K. pneumoniae* and other *Enterobacterales* pathogens. We demonstrate successful enrichment of target sequences using DNA extracted from (i) bacterial isolates, (ii) artificial isolate mixtures, and (iii) spiked human fecal samples. In addition, we provide an effective computational workflow for obtaining key characterization outcomes, including sequence type (ST) and AMR allelic variants, after sequencing.

## RESULTS

### CRISPR-Cas9 guides showed high conservation for target genes and species

**Fig 2:**
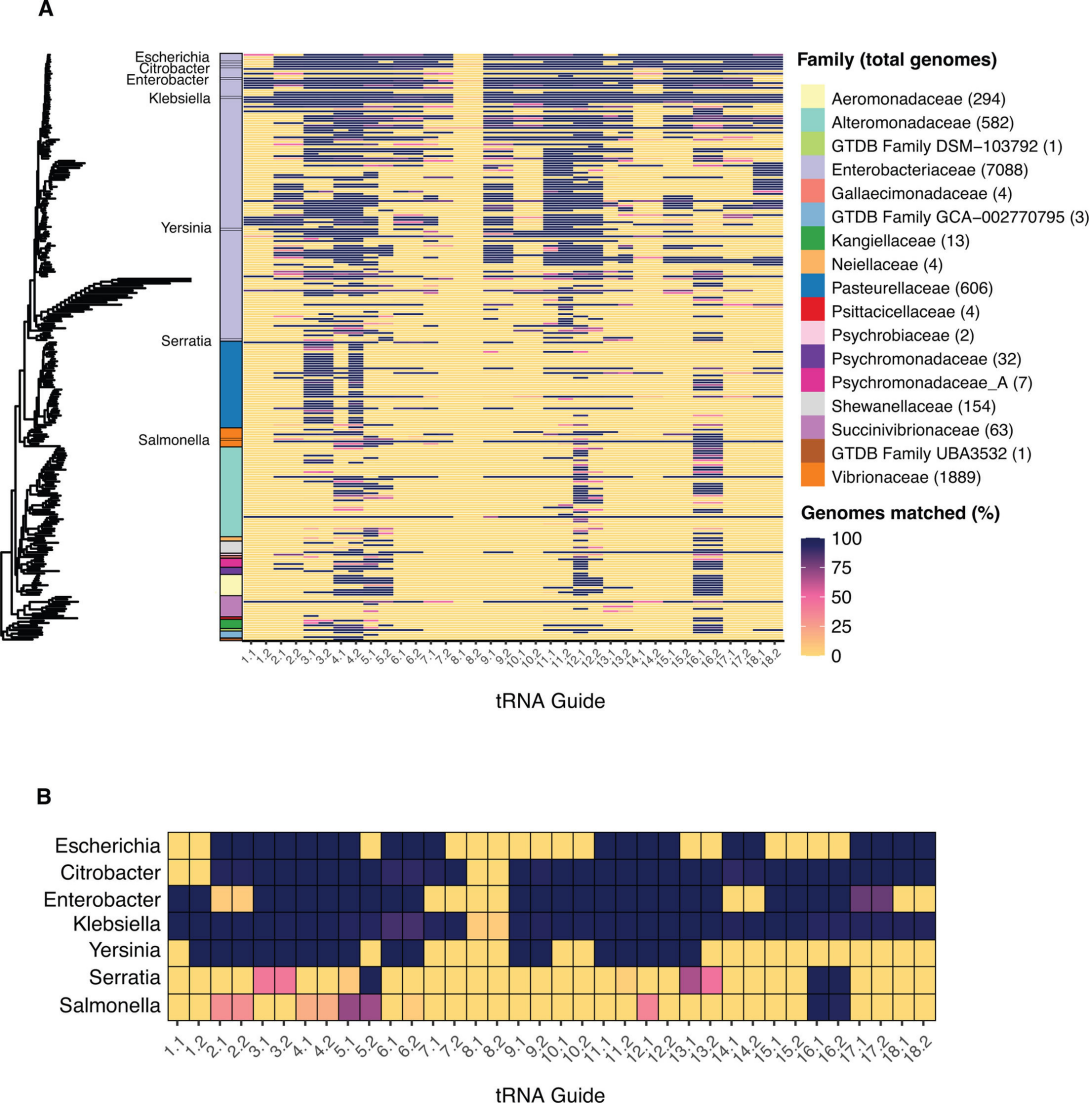
Conservation of tRNA guides across *Enterobacterales*. (**A**) Neighbour-joining tree of representative genomes from all genera in GTDB R95 classified as *Enterobacterales* (one genome per genus, *n* = 250 genera). The color spectrum of the heatmap shows the proportion of genomes matched to guide sequences in a dereplicated version of the full GTDB database for each genus (*n* = 11,339 total *Enterobacterales* genomes, genome count for each family shown in brackets). The color bar to the left of heatmap shows the GTDB-defined family of each genus. (**B**) Guide conservation in notable *Enterobacterales* pathogens.

To facilitate the enrichment of *Enterobacterales* pathogens, we selected 18 tRNA gene sequences from *K. pneumoniae* strain SGH10 (GenBank accession NZ_CP025080.1,) as enrichment targets. We chose tRNA genes due to their distribution around the chromosome and conservation within KpSC and *Enterobacterales*. Our CRISPR-Cas9 guides were designed using a pairing approach, comprised of two guides per gene conserved on opposing strands (*n* = 36 total tRNA guides) (Table S1; see Materials and Methods). To predict how our guides would perform in *Enterobacterales*, we aligned guides to all genomes classified as *Enterobacterales* in a dereplicated version of the Genome Taxonomy Database (GTDB) (version R95, *n* = 11,339 genomes; see Materials and Methods) (26). For each guide, we summarized the proportion of genomes per genus harboring an exact sequence match, which in principle enables targeted enrichment from the corresponding tRNA. One-third of genera (35.6%, 89 out of 250) contained at least half of the guide sequences in ≥75% of genomes. Meanwhile, 6.4% (16 out of 250) of genera contained 15 or more guide pairs in at least 75% of genomes (Fig. 2; Table S2). Most of these 16 genera were in the *Enterobacteriaceae* family, which contains multiple genera of clinical interest such as *Escherichia*, *Citrobacter*, *Enterobacter*, and *Klebsiella* (Fig. 2B). In *Klebsiella*, 34 out of 36 guides were highly conserved in 8 out of 11 species, including *K. pneumoniae* (Fig. S1). The *K. pneumoniae* strain that we originally designed guides for contained a rare variant of the Arg tRNA gene, so its corresponding guides (pair 8) were poorly conserved across *Enterobacterales*.

We sought to determine how specific to *Enterobacterales* bacteria our tRNA guides would be. Guide sequences were aligned to all reference genomes of the top 391 most observed GTDB species clusters in human gut samples according to recent metagenomic sequencing studies (Fig. S2) (see Materials and Methods) (27). Overall, we found our tRNA guides to be highly specific to *Enterobacterales*—33 out of 36 guide sites were conserved in one or zero species, and the remaining three guide sites were conserved in a maximum of 16 species (range 5–16, Fig. S2).

To enrich AMR genes, we designed a guide pair to target the *intI1* integrase gene, a part of the class 1 integron that mobilizes resistance genes and is commonly colocalized on plasmids with additional resistance determinants (see Materials and Methods) (28). We also designed four guide pairs to target highly conserved regions of the *bla*_IMP_, *bla*_OXA_, and *bla*_CTX-M_ extended-spectrum beta-lactamase (ESBL)/carbapenemase genes (see Materials and Methods). We prioritized targeting the alleles *bla*_IMP-4_, *bla*_OXA-48_, *bla*_CTX-M-14_, and *bla*_CTX-M-15_ as these are among the most commonly observed across Australian hospitals (25). However, we expected these guides to also enrich for other allelic variants. To determine this, we calculated exact matches between our guides and each allele present in the CARD database (see Materials and Methods) (29). Guide conservation varied according to allele variation in the AMR gene. For the less diverse genes *bla*_CTX-M_ and *bla*_IMP_, the guide sequences were conserved in in 77.4% (185 out of 232) and 40.2% (33 out of 82) of alleles, respectively (Fig. S3 and S4). The more diverse *bla*_OXA_ had exact matches to the guide sequences in just 3.9% (36 out of 912) alleles but was highly conserved in the *bla*_OXA-48_-like group of alleles known to confer carbapenem resistance (Fig. S5) (30).

Guide conservation rate increased when focusing on clinically important mobile carbapenemase/ESBL alleles (Table S3; see Materials and Methods). We found that guides targeted 84.6% (22 out of 26), 50% (5 out of 10), and 63.6% (7 out of 11) of mobile carbapenemase/ESBL alleles in *bla*_CTX-M_*, bla*_IMP_, and *bla*_OXA_, respectively. Alleles not targeted by guides were typically rarer—after adjusting for how frequently these alleles are observed in publicly available genomes, our guides were generally expected to target at least 90% of publicly available genomes possessing mobile carbapenemase/ESBL alleles (Table S3). These findings suggest that although our guides were not consistently conserved in every allele of every beta-lactamase family, they likely have a high rate of enrichment in mobile carbapenemase/ESBL alleles frequently observed in clinical settings.

Finally, we designed guides to target all seven *K. pneumoniae* MLST genes, as well as the *metG* gene proximal to the K locus in *K. pneumoniae*, to enable finer-scale typing. For MLST guides, we targeted regions of the gene outside the allele encoding section to preserve its entire sequence. We aligned our MLST guides to 11,446 *K*. *pneumoniae* publicly available genomes collected from 99 different countries over the last 100 years (see Materials and Methods) (31). Each guide pair matched to >99.6% of total genomes, with 89.9% (98 out of 109) of commonly observed STs containing a guide-matching sequence in all strains (Fig. S6). Our final guide pool comprised 31 pairs of guides (62 total) (Table S1).

### CRISPR-Cas9 guides consistently enriched for target sequences in *Enterobacterales* isolates

To assess guide performance we extracted DNA from 20 KpSC isolates, each with a publicly available completed genome and unique MLST and AMR gene profiles (Table S4). Nine isolates possessed an ESBL gene, and two possessed a carbapenemase gene. We performed CRISPR-Cas9 enrichment using our pool of 62 guides, followed by multiplexed ONT sequencing (see Materials and Methods). Reads were defined as on-target if they started within 20 bp of a target site, as this infers successful Cas9 cleavage and sequencing from these sites, and a guide was considered successful if the number of on-target reads was 10 times more than the background read depth estimated from off-target reads (see Materials and Methods).

**Fig 3:**
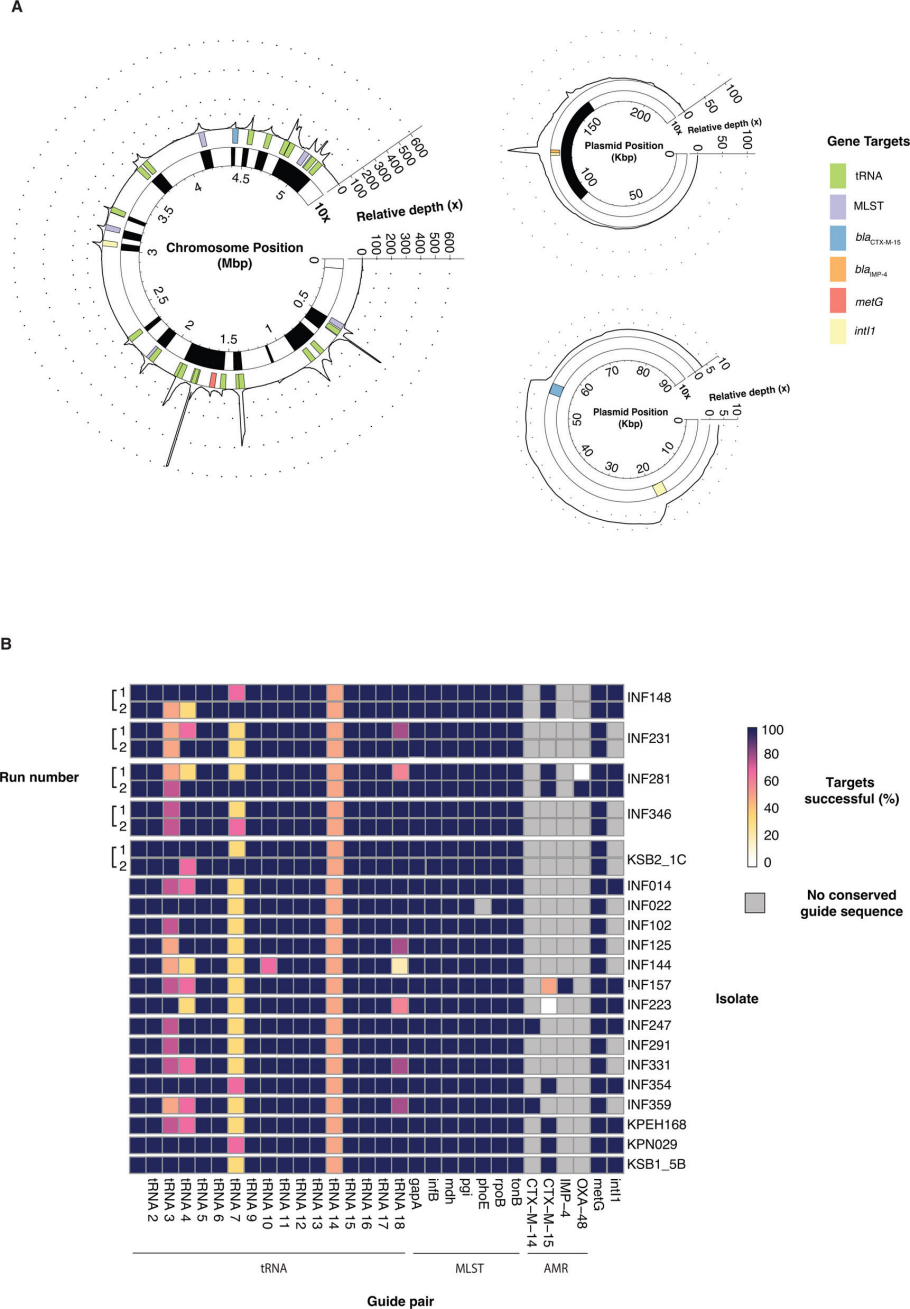
Guide pair performance in CRISPR-Cas9 enriched libraries of KpSC isolates. (**A**) Sequencing depth of all target contigs, for example, *K. pneumoniae* isolate INF157 following CRISPR-Cas9 enrichment. Gene target locations are shown as colored rectangles across the genome. Depth is shown relative to median depth of off-target alignments. The inner bar denoted as “10×” shows regions where relative depth is greater than 10 (shown in black). GenBank accessions of the target contigs are CP024528.1, CP024529.1, and CP024531.1 (**B**) Summary of guide performance across all 20 KpSC isolates. A successful target is defined as when the number of on-target reads is equal to or greater than 10× median depth of off-target reads. Run 1 refers to the initial sequencing with all 20 isolates, while run 2 refers to a repeat validation run on five randomly selected isolates.

On-target reads constituted a median of 86.7% (interquartile range [IQR] = 84.1–89.2%) of aligned reads across all isolates, and a median of 90.2% (IQR = 86.2–93.3%) of conserved guide targets per isolate were successful. Successful enrichment sites were characterized by large spikes in depth surrounding target genes, with minimal sequencing in untargeted regions likely resulting from random shearing of the DNA during library preparation (Fig. 3A; Fig. S7). A median of 30.0% (IQR = 24.1–33.5%) of each genome was recovered at above 10× depth relative to background depth, highlighting that targeting tRNA genes and using long reads can capture almost a third of the whole genome (Tables S5 and S6). All guide pairs with expected matches generated at least one successful enrichment target site in all but two isolates (*bla*_OXA-48_ in INF281 and *bla*_CTX-M-15_ in INF223; Fig. 3B). We replicated this experiment on five randomly selected isolates, and found similar enrichment performance, this time with a successful enrichment site for *bla*_OXA-48_ in INF281 (run 2; Fig. 3B).

To assess the capacity of CRISPR-enriched ONT data to determine ST and AMR allelic variants in these isolates, we used on-target reads to correct random draft alleles of our target MLST and AMR genes (see Materials and Methods). We found that 10 reads were sufficient to produce a correct MLST allele call in 81.8% (112 out of 137) of cases (Fig. S8). Accuracy continued to rise as depth increased, with 93.5% (87 out of 93) of alleles called correctly at 50× and 97.6% (40/41) correct at 100× depth. Overall, 80% (8 out of 10) of isolates with at least 30 on-target reads at each locus had a correct allele call in all seven MLST loci. Allele-level calls of AMR genes were comparably lower using this method, in part due to longer allele-encoding regions overall and a tendency for the majority of reads to travel from the cleavage site in a direction suboptimal to generating maximal coverage of the gene (Fig. S9). Despite this, seven out of nine isolates generated correct AMR alleles at nearly all depths. We were unable to reliably differentiate chromosomal and plasmid *bla*_CTX-M-15_ sequences following enrichment, as the surrounding ~6 kbp of chromosomal *bla*_CTX-M-15_ genes in tested isolates showed high similarity to known plasmid sequences (see Materials and Methods).

To determine whether our guide pair targeting the *intI1* integron integrase gene could identify colocalized AMR genes, we used an assembly-based approach (see Materials and Methods). We were able to detect over half of the AMR genes present on 70% (7 out of 10) of *intI1*-carrying plasmids by targeting either *intI1* or *intI1* plus one of our other AMR gene guides, highlighting the value of combining resistance-associated targets with long reads (Table S7; Fig. S10). Following a similar principle, we found that assembling on-target reads from the *metG* guide pair (the highly conserved gene upstream of the polysaccharide capsule-encoding K locus [see Materials and Methods] [32]) allowed us to generate a correct K locus call in 60% (12 out of 20) of *K. pneumoniae* isolates (Table S8; Fig. S11). The relatively low success of K locus calling was due to the distance between the K locus and *metG* (~57 kbp), which was the closest conserved sequence available to the target.

Despite the success of the paired guide pool (*n* = 62), it was not clear whether guide pairing was necessary for successful enrichment. To test this, we performed two additional enrichments using a single member of each pair rather than both (*n* = 31). Enrichment performance was lower across all metrics, with a median of 84.4% (IQR = 80.9–89.6%) of aligned reads coming from on-target regions and just 76.2% (IQR = 74.1–79.0%) of targets successful per isolate. These findings were validated with a smaller repeat experiment on 5 of the original 20 isolates, with individual guide performance also varying in the same isolate across repeat runs (Fig. S12; Table S9). For example, tRNA guides 3.1 and 2.2 were unsuccessful in all five isolates in the first run but successful in most isolates in the repeat run. Meanwhile, 4.2 and 7.2 were successful in the first run but were mostly unsuccessful in the repeat run. Overall, we found paired guides significantly outperformed unpaired guides by total targets successful [*X*^2^(1, *N* = 3,482) =65.9, *P* = 6.88 × 10^−16^] and number of libraries where every conserved guide had a successful enrichment site [*X*^2^(1, *N* = 79) =51.1, *P* = 5.29 × 10^−12^] (Table S10).

By designing guides to target tRNA genes that are highly conserved in *Enterobacterales*, we aimed to enrich several common pathogens in addition to *K. pneumoniae*. To validate this, we tested tRNA guides in a selection of *Enterobacterales* isolates not in the KpSC. Enrichment results were similarly successful to those observed in *K. pneumoniae* isolates, with 68.4–94.7% (26–36/38) of tRNA guides having a conserved sequence in each isolate, and each guide generating at least one successful enrichment site in isolates with a conserved sequence (Tables S11 and S12).

### CRISPR-Cas9 enrichment improved characterization of *K. pneumoniae* from human fecal samples compared to unenriched metagenomic sequencing

We then validated our CRISPR-Cas9 enrichment guides and methodology in complex patient samples, as this is their intended use-case. We spiked *K. pneumoniae* strain INF298 cells into three human fecal samples at two abundances (4 × 10^6^ and 4 × 10^7^ CFU/g, see Materials and Methods). We performed quantitative PCR (qPCR) on all samples to determine baseline *K. pneumoniae* DNA abundance and confirm successful spike-ins (see Materials and Methods). Unspiked samples were estimated to contain 0.01–0.1% abundance of native *K. pneumoniae*, while spiked samples were estimated at 0.3–3.7% abundance (Table S13).

**Fig 4:**
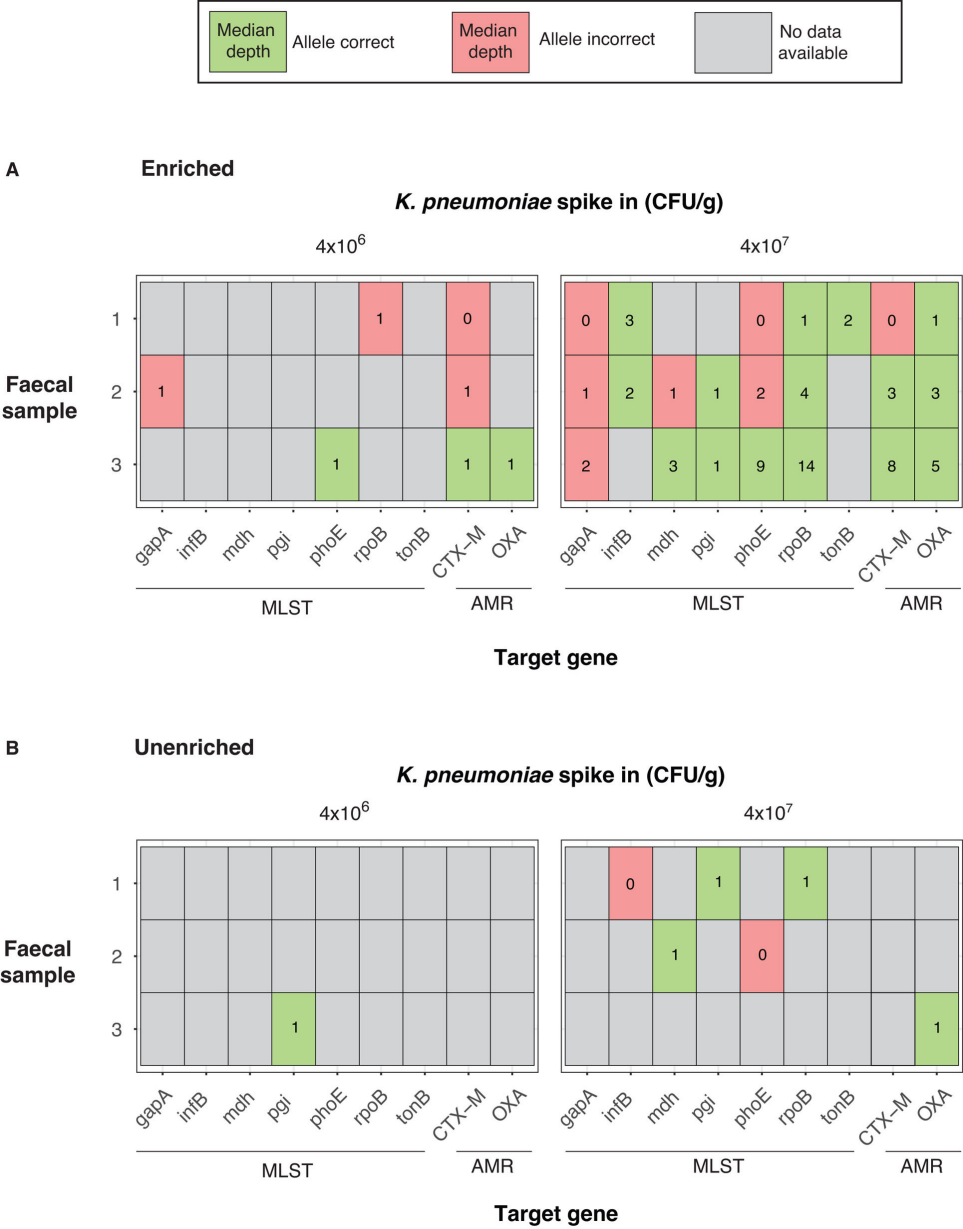
Allele call accuracy of target AMR and MLST genes following enriched and unenriched sequencing of human fecal samples spiked with *K. pneumoniae* strain INF298 (Refseq accession GCA_904864465.1) at 4 × 10^6^–4 × 10^7^ CFU/g. Median depth was calculated from the total number of reads aligning to the target gene. (**A**) Allele call accuracy following CRISPR-Cas9 enriched sequencing. (**B**) Allele accuracy following unenriched sequencing.

Both enriched and unenriched libraries led to detection of *K. pneumoniae* in all aliquots, typically with comparable amounts of *K. pneumoniae-*classified bases sequenced and coverage of the spiked strain genome (Table 1). Enriched libraries generated more *K. pneumoniae* reads aligning to MLST genes than unenriched libraries in every case, with several unenriched libraries not containing any MLST reads at all. After polishing draft sequences of MLST genes with aligned reads, enriched libraries of 4 × 10^7^ CFU/g spiked aliquots generated correct allele calls in 47.6% (10 out of 21) of loci compared to 14.3% (3 out of 21) in unenriched libraries. 4 × 10^6^ CFU/g appeared to be beneath the limit of consistent MLST characterization, with correct calls in just 4.8% (1 out of 21) loci across enriched and unenriched libraries (Fig. 4). CRISPR-Cas9 enrichment was even more effective at picking up target AMR genes, with 55 reads aligning to AMR genes across enriched libraries compared to one read across all unenriched libraries (Table 1). This led to seven correct AMR allele calls in enriched libraries versus one correct AMR call in unenriched libraries (Fig. 4). For 10 out of 12 spiked aliquots, enrichment led to detection of target AMR genes within the first 10 h of sequencing, with two aliquots (samples 2 and 3, 10^7^ CFU/g) obtaining detection of target AMR genes within the first hour (Table S14; Fig. S13). All AMR reads consistently aligned to the flanking regions in the reference strain (Fig. S14). When also considering that unspiked aliquots did not generate any target AMR reads, it is highly likely that the *bla*_CTX-M-15_ and *bla*_OXA-48_ reads originated from the INF298 spike in strain. Cas9 cleavage and sequencing usually led to reads in both directions from the cleavage site, but occasionally all on-target reads traveled in a single direction, leading to poor AMR gene coverage and an incorrect allele call (Fig. S14; Table S14). While most enriched aliquots with on-target AMR reads generated 86% or higher coverage of the target gene, in some cases, just 10–13% of *bla*_CTX-M-15_ was sequenced due to a combination of low overall yield and on-target reads traveling in a direction suboptimal to full coverage (sample 1, 4 × 10^7^ CFU/g, enriched, Fig. S14C).

**TABLE 1:** Sequencing and enrichment statistics following CRISPR-Cas9 enriched and unenriched sequencing of human fecal samples spiked with varying abundances of *K. pneumoniae* strain INF298 (Refseq accession GCA_904864465.1)

Sample	*K. pneumoniae* spike in (CFU/g)	*K. pneumoniae* relative abundance (qPCR) (%)	Enrichment	Library input (ng)	Sequencing duration (h)	*K. pneumoniae*-classified bp (per ng DNA input)	*K. pneumoniae* strain INF298 genome coverage (% bases > 0× depth)	*K. pneumoniae* MLST reads	*bla*_CTX-M-15_ reads	*bla*_OXA-48_ reads	Class 1 integron coverage (% ≥1× depth)
1	0	0.11	Enriched	84	10	161	0.17	0	0	0	0.0
1	0	0.11	Unenriched	20	10	100	0.01	0	0	0	0.0
1	4 × 10^6^	0.31	Enriched	78	10	1,669	1.97	1	1	0	4.5
1	4 × 10^6^	0.31	Unenriched	12	10	1,231	0.2	0	0	0	0.0
1	4 × 10^7^	2.14	Enriched	90	40	7,280	5.63	10	3	5	13.9
1	4 × 10^7^	2.14	Unenriched	108	40	14,191	16.16	3	0	0	0.0
2	0	0.01	Enriched	48	10	66	0	0	0	0	0.0
2	0	0.01	Unenriched	66	10	40	0	0	0	0	0.0
2	4 × 10^6^	0.27	Enriched	132	10	1,115	2.28	2	1	0	12.6
2	4 × 10^6^	0.27	Unenriched	66	10	1,478	1.19	0	0	0	0.0
2	4 × 10^7^	2.02	Enriched	78	40	11,005	7.7	13	6	6	98.6
2	4 × 10^7^	2.02	Unenriched	84	40	10,338	10.71	2	0	0	0.0
3	0	0.07	Enriched	78	10	94	0	0	0	0	0.0
3	0	0.07	Unenriched	66	10	349	0.02	0	0	0	0.0
3	4 × 10^6^	0.69	Enriched	102	10	1,442	2.01	2	1	3	17.2
3	4 × 10^6^	0.69	Unenriched	102	10	4,763	5.18	1	0	0	0.0
3	4 × 10^7^	3.70	Enriched	108	40	20,639	14.91	40	17	13	95.3
3	4 × 10^7^	3.70	Unenriched	108	40	18,869	24.45	0	0	1	13.9

**Fig 5:**
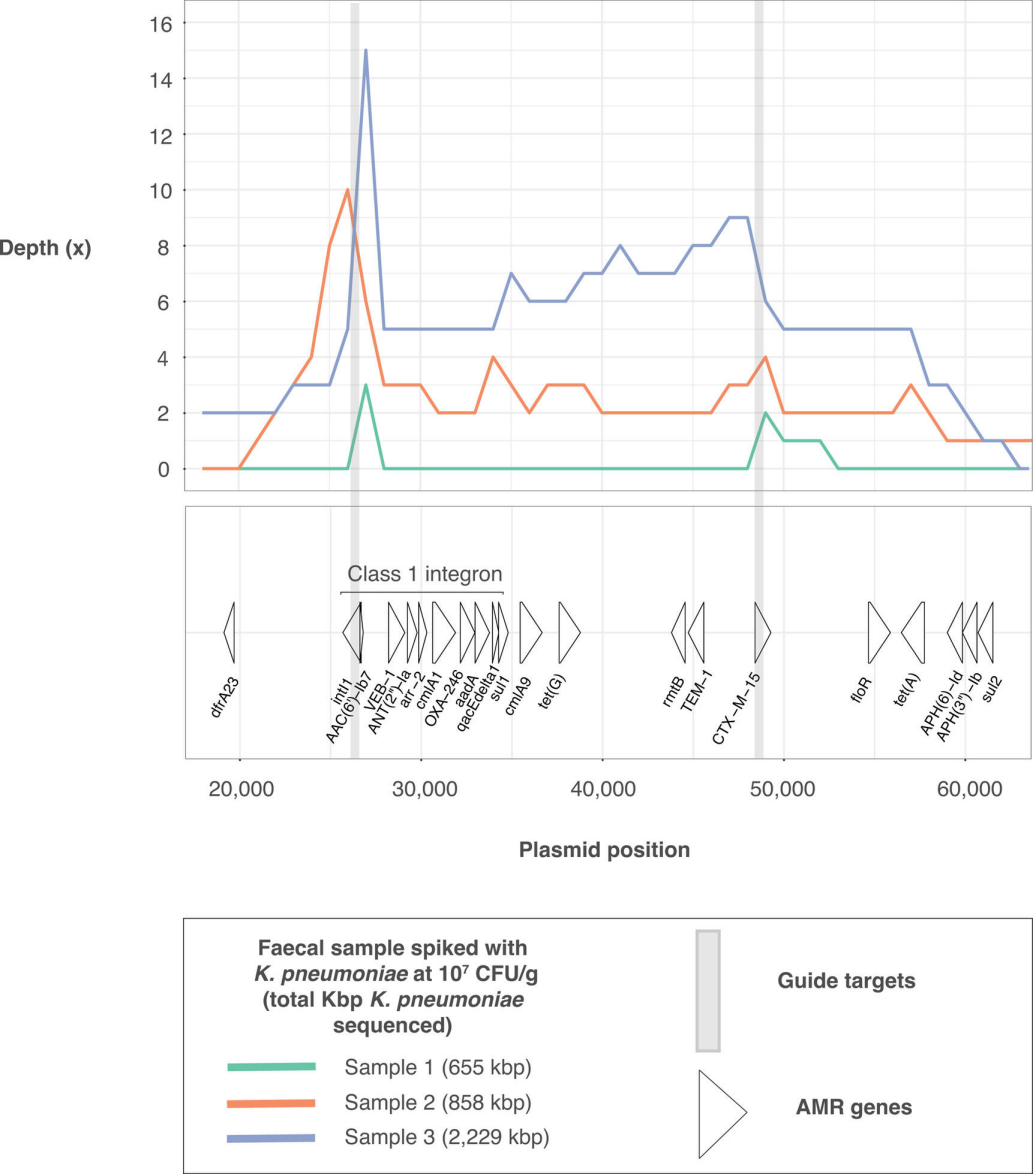
Depth of sequencing of *K. pneumoniae* strain INF298 plasmid (GenBank accession CP110595.1) following sequencing of three human fecal samples spiked with INF298 at 4 × 10^7^ CFU/g. The bottom panel is labeled with the regions of all AMR genes on the plasmid, with the commonly observed class 1 integron labeled.

Enriched runs were able to characterize the class 1 integron present on a plasmid of the spiked strain, compared with unenriched runs which generated zero *Klebsiella-*classified reads aligning to the integron, except in one aliquot (sample 3, 10^7^ CFU/g; Table 1). *Klebsiella-*classified reads from spiked, enriched aliquots generated 4.5–98.6% (median 15.5%) coverage of the class 1 integron, with large spikes in depth at *intI1* (Table 1; Fig. 5). While enriched runs generated two and three reads aligning to the *metG* target gene in samples 2 and 3 spiked with 10^7^ CFU/g respectively, no *Klebsiella-*classified reads aligned to the INF298 K locus in any enriched aliquots. This is likely due to the *metG* gene being 57,219 bp away from the start of the K locus in this strain, requiring extremely long reads for effective characterization. Unenriched aliquots generated no *Klebsiella-*classified reads aligning to the K locus except for the 4 × 10^7^ CFU/g spike-ins, which generated one aligned read each. These reads provided limited information about the locus, with coverages of 2.4%, 7.8%, and 21.3% for samples 1–3, respectively.

### Enrichment performance is highly correlated with guide conservation

To better understand how enrichment of *K. pneumoniae* was affected by guide conservation in non-target species present in a complex sample, we generated four replicate mock microbial community mixtures consisting of DNA from 11 different bacterial species at equimolar amounts, with *K. pneumoniae* strain INF298 DNA spiked in at 0%, 0.08%, 0.8%, and 8% abundance (see Materials and Methods). Isolates in the mock community showed varying amounts of guide conservation, ranging from 0 to 46 conserved sequences (Table S15). Following CRISPR-Cas9 enrichment and sequencing, we classified reads as originating from a given species using an alignment-based approach (see Materials and Methods). We then performed similar characterizations of the INF298 strain as in the fecal samples, using reads assigned to *K. pneumoniae*. We found trends to be very similar, with limited enrichment of *K. pneumoniae*-assigned reads (normalized to input abundance), but with high output of data from target genes (Table S16; Fig. S15). While MLST, AMR, and K locus calls were inconsistent at the 0.08–0.8% input abundance, as expected nearly all alleles were correct following enrichment and sequencing of the mixture spiked with 8% *K*. *pneumoniae* (Table S16).

**Fig 6:**
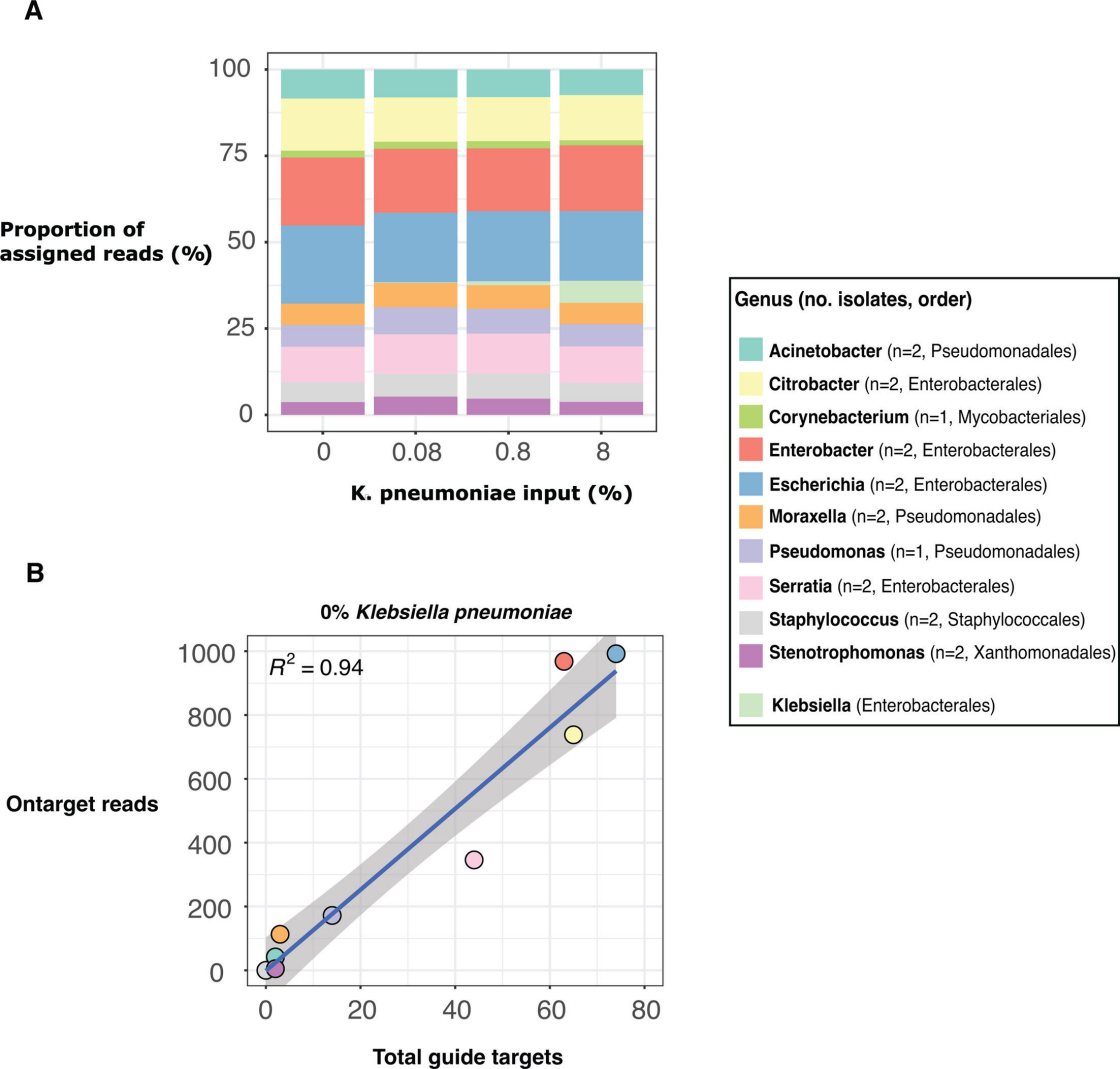
Relationship between guide conservation and enrichment performance following CRISPR-Cas9 enriched sequencing of a mock microbial community with varying amount of *K. pneumoniae* strain INF298 (Refseq accession GCA_904864465.1) DNA. (**A**) Taxonomic distribution of sequencing output based on an alignment-based approach (see Materials and Methods). (**B**) Linear regression analysis between the total guide targets and total on-target reads for isolates of a given genus following sequencing of the mock community with no *K. pneumoniae* included.

As the mock microbial mixture comprised completed genomes, we could determine the relationship between guide conservation and performance (see Materials and Methods). We found the number of conserved guide targets to be highly predictive (*R*^2^ = 0.90–0.94) of the amount of on-target sequence data assigned to each genus (Fig. 6; Fig. S16; Tables S15 and S17).

Similarly, our fecal sequence data included on-target reads generated from taxa other than the spiked *K. pneumoniae* strain. As expected, the majority of tRNA reads were classified as originating from Proteobacteria genera such as *Klebsiella* and *Escherichia* (Fig. S17). However, 27 reads were classified to the phylum of Bacteroidota, all of which aligned to tRNA-Gly. Guide pair 3 (targeting tRNA-Gly) was predicted to be conserved in many Bacteroidota species according to our initial conservation analyses, clarifying that these non-Proteobacteria tRNA sequences were in fact a by-product of our guide design rather than off-target cleavage and enrichment (Fig. S2 and Fig. S17).

## DISCUSSION

By using CRISPR-Cas9 enrichment to target highly conserved tRNA genes, we selectively enriched and sequenced large sections of pathogen genomes, using DNA extracted from pure *K. pneumoniae* and other *Enterobacterales* specie*s* isolates, artificial isolate mixtures, and human fecal samples (Fig. 2 and 3; Table 1; Fig. 6). We generated allele-level MLST and AMR calls by targeting highly conserved regions of these loci, and utilized long reads to identify their genetic context and ensure that they originated from the target strain (Fig. 4 and 5; Fig. S14). We found CRISPR-Cas9 enrichment can outperform traditional metagenomics by identifying low abundance MLST and AMR genes from human fecal samples, with enriched libraries often picking up genes that went completely undetected in unenriched libraries (Table 1). Finally, we demonstrated improved enrichment consistency when using a paired guide approach, wherein multiple guides targeted overlapping regions on opposite strands of a target gene (Fig. S12; Table S9). Our study is the first bacterial application to be validated in human fecal samples, enrich for tRNA genes highly conserved across *Enterobacterales* and benchmark analysis methods for identifying allelic variants from the resulting sequence data.

Although most guides generated successful enrichment in isolates with a conserved sequence, we observed large variability in depth surrounding target regions (Fig. 3B; Fig. S7). While factors, such as GC content, secondary structure formation, and DNA methylation, have been cited as factors that may influence Cas9 cleavage success, we find this unlikely to be applicable to our context due to varying performance on the same DNA extracts over repeated sequencing runs (Table S9) (33–40). Our results indicate that designing two guides targeting overlapping regions in the target gene can mitigate some of these differences in performance and improve enrichment consistency. However, it is unclear whether improved performance was specifically due to their overlapping target sites or simply increasing the number of guides targeting the gene. A pairing approach may be best for critically important target genes such as MLST and AMR, while one guide may be sufficient for non-essential targets. Guide conservation was highly predictive of enrichment performance in complex samples, resulting in some enrichment of Bacteroidota sequences from a tRNA guide pair (Fig. S2 and S17). Future studies using this guide pool to enrich *Enterobacterales* from fecal samples may wish to exclude this guide pair to avoid unintended enrichment of non-target species—guides can be easily included and excluded as desired.

This study sought to explore how CRISPR-enriched sequence data can be used for pathogen detection and typing. Our results suggest that the presence of *K. pneumoniae* MLST loci could be a reliable indicator of the presence of this species. Furthermore, the majority of MLST allelic variant calls were correct, enabling the detection of specific lineages. Enrichment of AMR gene targets was also reliable, although a higher rate of incorrect allele calls was observed than for MLST loci (Fig. S8 and S9). This is likely due to the larger size of AMR genes such as *bla*_IMP_, *bla*_OXA_, and *bla*_CTX-M_, increasing the chances of inaccuracies when generating consensus sequences. Position of the guide sequence may also play a role—while MLST genes had guide target positions outside the region that specifies an allele, the entire AMR gene is allele encoding and thus will be disrupted regardless of the position of the guide sequence. While targeting within the AMR gene means that no single on-target read will contain the entire sequence, it enables greater consistency in enrichment across a variety of genetic contexts. Finding targets in highly conserved flanking regions to preserve the entire AMR gene is impossible in many cases, particularly in highly variable plasmid sequences. We also found that a lack of overall gene coverage was the limiting factor in most incorrect allele calls, as opposed to disruption in the alignments (Fig. 4; Table S14; Fig. S14). Genes with guide targets closer to their ends may have more varied coverage than genes with guide targets around the middle, particularly at lower depths with a small number of on-target reads. While most on-target reads will cover approximately half of the target gene if the guide targets are in the middle of the gene (Fig. S14B), if targets are closer to the end they could cover nearly the entire gene (sample 2, 4 × 10^6^ CFU/g, enriched, Fig. S14C) or a small fraction of the gene (sample 1, 4 × 10^7^ CFU/g, enriched, Fig. S14C) depending on which direction the on-target read travels.

While we were able to generate accurate MLST and AMR allele calls in many cases throughout this study using ONT’s R9.4.1 sequencing chemistry, the latest R10.4.1 ONT flowcells provide much greater read-level and consensus base-call accuracy (41) and would thus presumably yield substantially higher accuracy even with comparable amounts of data.

Yield of target sequences could be further improved via the removal of non-cleaved molecules prior to sequencing. This would allow molecules with sequencing adapters attached to move more freely into the pores of the flowcell rather than being blocked by the abundance of non-target molecules still present in the solution. Some efforts have been made to improve enrichment efficiency through endonuclease depletion of untargeted DNA fragments in eukaryotic applications (42). However, this approach is not as useful for bacterial applications where the genetic context of a target is often unknown, as it requires enrichment sites at both ends of the target region (42). Future studies looking to improve enrichment efficiency for the single excision approach shown here could focus on molecular methods for binding to and extracting molecules with terminal phosphate groups. Meanwhile, increasing the number of guides may help to further increase the ratio of cleaved to non-cleaved DNA fragments. Finally, they could also perform several DNA extracts or non-specific PCR to increase input yield and sequence one sample at a time, as opposed to the multiplexed method used in this study.

Another major benefit to our ONT CRISPR-Cas9 enrichment protocol is the reduction of computational requirements compared to deep metagenomic sequencing. While typical metagenomics can often require multiple days of sequencing to reliably detect low abundance AMR genes, our CRISPR-enriched libraries detected targeted *bla*_CTX-M-15_ and *bla*_OXA-48_ genes in human fecal samples from as little as one hour of sequencing. When looking at the fecal data overall, we found CRISPR-Cas9 enrichment generated 11.3× the amount of MLST reads and 56× the amount of AMR reads while using 3.3× less storage space (16 GB vs 53 GB) compared to unenriched sequencing of the same samples (Table 1). These reductions in computational requirements could substantially improve the cost-effectiveness and feasibility of sequencing directly from patient samples in clinical settings. This is particularly important in contexts where resource constraints preclude traditional metagenomic approaches and low-cost ONT sequencing equipment predominates.

While this study demonstrated the improved performance of CRISPR-Cas9 enrichment over typical metagenomic approaches, there are scenarios where other enrichment methods may be more appropriate. In some contexts where there is no knowledge of the disease-causing pathogen, approaches based on background depletion, such as saponin depletion, CpG methylated DNA removal, and depletion by hybridization, may be more suitable. While relatively straightforward and affordable, the CRISPR-Cas9 enrichment approach also requires more planning and experimental validation than computational enrichment approaches such as ONT’s adaptive sampling. While the performance of adaptive sampling has been limited so far, it may be a more accessible method for where this development is not feasible. Meanwhile, although this study displayed highly successful enrichment of target pathogens, it was limited to testing in three human samples. Future studies may look to validate performance across a larger number of human samples and a wider variety of specimen types. While a focus of analyzing fecal data were validating the origin of target reads, this may not be necessary for less complex sample types such as blood, cerebrospinal fluid, and urine.

Our findings indicate that CRISPR-Cas9-based enrichment shows promise for targeted long-read sequencing of bacteria from clinical samples. This approach enables rapid and culture-free surveillance screening of patient samples for problematic pathogens, including *K. pneumoniae*. The additional information provided by sequencing data could inform control strategies or identify patients colonized with high-risk strains.

## MATERIALS AND METHODS

### CRISPR-Cas9 guide design

To enable the detection of the widest range of MLST, beta-lactamase, *intI1*, and *metG* alleles as possible, we obtained a large collection of alleles for each targeted gene to identify highly conserved sequences. For the beta-lactamase genes, this collection consisted of all alleles present in a curated version of the Comprehensive Antibiotic Resistance Database (CARD) as of May 2020 (*n* = 912 alleles for *bla*_OXA_, *n* = 232 for *bla*_CTX-M_, and *n* = 82 for *bla*_IMP_) (29, 31). For MLST genes, the gene sequence present in strain SGH10 was aligned using BLASTn v2.13.0 (43) to a large collection of dereplicated publicly available KpSC genomes (*n* = 11,446) and all full-length matches to the query were retained (31). This data set included genomes from 99 countries collected from animal, environmental, food, and human sources over the last 100 years (31). *intI1* alleles were identified by aligning the publicly available reference gene (GenBank accession CP024557.1) to the same KpSC genome collection and retaining full-length matches. *metG* alleles were identified using panaroo v1.2.2 (44) from a set of 328 KpSC genomes collected between 2013 and 2014 from the Alfred Hospital in Melbourne, Australia (45–47).

All alleles from each target gene (with the exception of *metG*, where we utilized the alignment from panaroo) were aligned using MUSCLE v3.8.31 (48). Highly conserved sequences were visually identified in Jalview v2.11.1 (49). For MLST genes, we ensured that the conserved regions selected were outside of the MLST allele-coding region to facilitate MLST typing. All conserved regions were input into the CRISPR-Cas9 guide design tool CHOPCHOP v3 (33) using the “nanopore enrichment” setting, with *Homo sapiens* hg38/GRCh38 as the background organism to minimize potential matches to human DNA. Preference was given to guides with minimal close matches to the background genome (MM1 = 0, MM2 <3, and MM3 <5), %GC ranging from 40% to 60%, and no self-complementarity. Based on preliminary results, we hypothesized that designing two guides with conserved regions on opposite strands of target genes would be more effective than a single guide per target sequence. To test this, we designed guides with these overlapping regions for each target gene. If the “nanopore enrichment” setting did not yield two guides with overlapping regions, we used the default “knock-out” setting to produce a larger pool of candidate sequences. We ordered 31 guide pairs (*n* = 62 total) guides from Integrated DNA Technologies using the Custom Alt-R CRISPR-Cas9 guide RNA tool Table S1.

### Analysis of guide conservation

To assess tRNA guide conservation within *Enterobacterales*, we first prepared a dereplicated version of *Enterobacterales* genomes in the Genome Taxonomy Database (GTDB) (release 95) (26). Each GTDB species was dereplicated with Assembly-Dereplicator v0.1.0 (50), first using a distance threshold of 0.001, then increasing the threshold until either the number of assemblies dropped below 100 or the threshold reached 0.05 (*n* = 11,339 total *Enterobacterales* genomes) . We then aligned tRNA guides to all *Enterobacterales* genomes using bowtie2 v2.3.5.1 (51) and summarized the proportion of genomes in each genus with a perfectly conserved guide . To visualize genera in *Enterobacterales* and *Klebsiella*, a single representative genome was taken from each genera or species respectively and used as input into mashtree v1.2.0 (52). For conservation outside *Enterobacterales,* guides were aligned to all genomes of the top 500 most commonly observed GTDB (release 89) species in human gut samples (27) using bowtie2 v2.3.5.1 (51). Species clusters that were unclassified (*n* = 91), duplicated (*n* = 5), or members of *Enterobacterales* (*n* = 13) were excluded from a final data set of 391 species clusters.

AMR guides were aligned to all alleles of each target gene present in the CARD database as of June 2022 using bowtie2 v2.3.5.1 (29, 51). Multiple sequence alignment and BioNJ trees of all alleles for each gene were generated in seaview (53, 54). Mobile carbapenemase/ESBL alleles were defined in this study as those found in multiple *Enterobacterales* species according to CARD prevalence data. Prevalence data were then used to summarize their rate of carriage in public assemblies and the rate at which those assemblies contain conserved guide sequences. MLST guides were aligned to the previously described database of 11,446 *K*. *pneumoniae* genomes (31) using bowtie2 v2.3.5.1 (51). All conservation calculations were performed in R.

### Sample preparation

Bacterial isolates were grown overnight on Luria-Bertani (LB) agar and DNA extraction was performed using the GenFind v3 gDNA extraction kit according to standard protocol (Table S4) (Beckman Coulter). For the mock microbial community, we pooled equimolar amounts of genomic DNA from 18 bacterial isolates (Table S14). *K. pneumoniae* strain INF298 (Refseq accession GCA_904864465.1) genomic DNA was spiked into four duplicate aliquots of the community at varying relative abundance (0%, 0.08%, 0.8%, and 8%).

For fecal experiments, we mixed 0.3 g from each sample in 1 mL of sterile 1× phosphate-buffered saline to ensure even bacterial distribution. We then took three aliquots (0.1 g faeces each) of each sample and spiked in 0, 4 × 10^5^, or 4 × 10^6^ CFU of *K. pneumoniae* strain INF298 cells grown overnight in LB broth. This resulted in 0.1 g fecal aliquots with *K. pneumoniae* strain INF298 spiked in at concentrations of 0, 4 × 10^6^, and 4 × 10^7^ CFU/g; an estimated range that would typically be found in fecal samples (2, 55, 56). We extracted fecal DNA using the “three peaks” method to retain long DNA fragments for Oxford Nanopore sequencing (57). Briefly, this involves first removing free DNA present in the sample, then enzymatic cell lysis and DNA extraction, followed by bead beating and DNA extraction.

### Quantitative PCR

To determine *K. pneumoniae* abundance in fecal DNA extracts, qPCR was conducted using Promega’s GoTaq reagents with primers specific to *K. pneumoniae* and a standard curve of pure INF298 genomic DNA (58). Sample, reagent, and thermocycler details can be found in Table S13.

### CRISPR-Cas9 enrichment and DNA sequencing

CRISPR-Cas9 enrichment was performed according to Oxford Nanopore’s Cas9 Targeted Sequencing protocol with some modifications to facilitate multiplexed libraries (Methods S1). Briefly, this involved preparing Cas9 ribonucleoproteins (RNPs) by combining *Streptococcus pyogenes* Cas9 nuclease, tracrRNA, and crRNAs. Genomic DNA was then dephosphorylated using calf intestinal phosphatase to prevent adapter ligation. Cas9 cleavage was induced at target sites to expose DNA terminal phosphate groups and allow for adapter ligation in these areas (Fig. 1). Final DNA libraries were prepared using ligation kit LSK-109 and the barcoding expansion kit EXP-NBD196, sequenced on R9.4.1 MinION flowcells and basecalled using the super model of guppy v6.2.1 for isolate experiments and v7.1.4 for fecal experiments. For fecal experiments, we delayed pooling barcodes until after adapter ligation to minimize any cross-barcode leakage (Methods S2). Enriched and unenriched libraries of each fecal sample were run for the same duration (10 h for 0 CFU/g and 4 × 10^6^ CFU/g aliquots, 40 h for 4 × 10^7^ CFU/g aliquots) using separate flowcells with comparable pore counts.

### Analysis of enrichment success

For isolate and mock community experiments, we used previously completed genomes (Tables S4 and S15). Guide sequences were aligned to assemblies using bowtie2 v2.5.1 with the -a parameter to identify all target regions (51). Reads were then aligned to assemblies using minimap2 v2.24 (59) with the -map-ont, -c and --secondary=no parameters . After aligning CRISPR-enriched sequence data to the completed genome of each isolate, on-target reads were defined as those with alignments starting or ending near the conserved guide site, as these alignments are most likely the result of Cas9 cleavage, adapter ligation and sequencing beginning at these sites. We allowed up to 20 bp of flexibility in the start locations of these alignments to account for known instances of untrimmed adapters and poor sequence quality at the termini of ONT reads (60). Successful guide sites were defined as those with 10 or more on-target reads divided by the median depth of off-target (non-on-target) reads for that contig. Normalizing on-target read levels to the depth of off-target reads was to account for yield differences between isolates. To visualize enrichment performance, the depth of sequencing at each position in the genome was calculated using samtools depth v1.1.7 (61). Circular depth plots were generated using the R package circlize v0.4.10, while linear depth plots were generated in ggplot2 (62). Chi-squared tests to compare paired and unpaired guide performance were generated in R using the chi.test function with default settings.

To assign reads to species following CRISPR-Cas9 enrichment and sequencing of the mock microbial community, we first aligned reads to each of the isolates in the mixture. We then classified them as originating from a given species if the highest scoring alignments for at least 80% of positions in the read stemmed from isolates of that species. Linear regression analysis to assess the relationship between guide conservation and performance in the artificial bacterial mixture was performed using the ggpmisc package. A *P* value less than 0.05 was treated as statistically significant.

For fecal experiments, we performed species classification using Kraken 2 with the GTDB database release 202 as reference (63, 64). To extract reads classified into taxons of interest, we used read IDs from the Kraken 2’s output as input for seqtk’s subseq command (https://github.com/lh3/seqtk). To determine the number of reads aligning to target loci, we aligned reads to fasta files of the target genes using minimap2 with the -c and --secondary=no flags. To extract read according to sequencing duration, we sorted fastq read IDs by the start_time flag on the header line and calculated the elapsed time since the first read of the run. We then used the read IDs that were beneath a given sequencing duration as input into seqtk’s subseq command (https://github.com/lh3/seqtk).

### Characterising MLST and AMR genes using enriched sequences

We generated consensus sequences of target MLST and AMR genes by using raw reads to polish a random allele known not to match the allele present in each genome using medaka v1.5.0 (https://github.com/nanoporetech/medaka). For MLST genes, the draft sequence was a randomly chosen allele and for AMR genes, we chose a random allele from the same clade as the target allele. We found that changing the draft allele within these parameters had no noticeable effect on consensus accuracy.

To determine whether we could differentiate between chromosomal and plasmid *bla*_CTX-M_ reads following CRISPR-Cas9 enrichment and sequencing of *K. pneumoniae* isolates, we classified reads using Kraken 2 (63) with a custom database of *Enterobacterales* chromosomes and plasmids (65). The database included all fully assembled KpSC chromosomes found in NCBI and all complete *Enterobacterales* plasmids in PLSDB as of December 2023 (66).

To determine how many *intI1*-adjacent AMR genes we could identify in isolate experiments, we assembled all on-target reads from each *intI1*-containing plasmid using flye v2.9 (67) with 70,000 as the --genome-size parameter . We ran the resulting assemblies through Kleborate v2.3.2 (31) with the -r parameter and compared AMR results to those run on the plasmid from the completed assembly . For *metG* analyses, we assembled on-target *metG* reads using flye v2.9 (67) with 70,000 as the --genome-size parameter, ran Kaptive v3.0.0b (32, 68) and compared results to the completed assembly. For fecal experiments, we extracted reads that aligned to the *intI1* gene and aligned those to the June 2022 version of the CARD AMR database (29).

## Data Availability

All sequence data generated in this study have been deposited in the NCBI database under the BioProject accession PRJNA1123839.

## SUPPLEMENTAL MATERIAL

Methods S1 msystems.01413-24-s0001.docx Basic CRISPR-Cas9 enrichment protocol.

Methods S2 msystems.01413-24-s0002.docx CRISPR-Cas9 enrichment protocol focused on minimising barcode leakage.

Figures S1 to S6 msystems.01413-24-s0003.pdf Supplemental figures.

Figure S7 msystems.01413-24-s0004.pdf Depth plots of all enriched isolate sequencing.

Figures S8 to S17 msystems.01413-24-s0005.pdf Additional supplemental figures.

Supplemental Tables msystems.01413-24-s0006.xlsx Tables S1 to S17.

ASM does not own the copyrights to Supplemental Material that may be linked to, or accessed through, an article. The authors have granted ASM a non-exclusive, world-wide license to publish the Supplemental Material files. Please contact the corresponding author directly for reuse.
