# Supplementary material for: Targeted sequencing of Enterobacterales bacteria using CRISPR-Cas9 enrichment and Oxford Nanopore Technologies
Source: mSystems. 2025 Jan 8;10(2):e01413-24. doi: 10.1128/msystems.01413-24 (PMC11834407; doi:10.1128/msystems.01413-24)
Supplement: Figures S1 to S6 — Supplemental figures. [file msystems.01413-24-s0003.pdf]

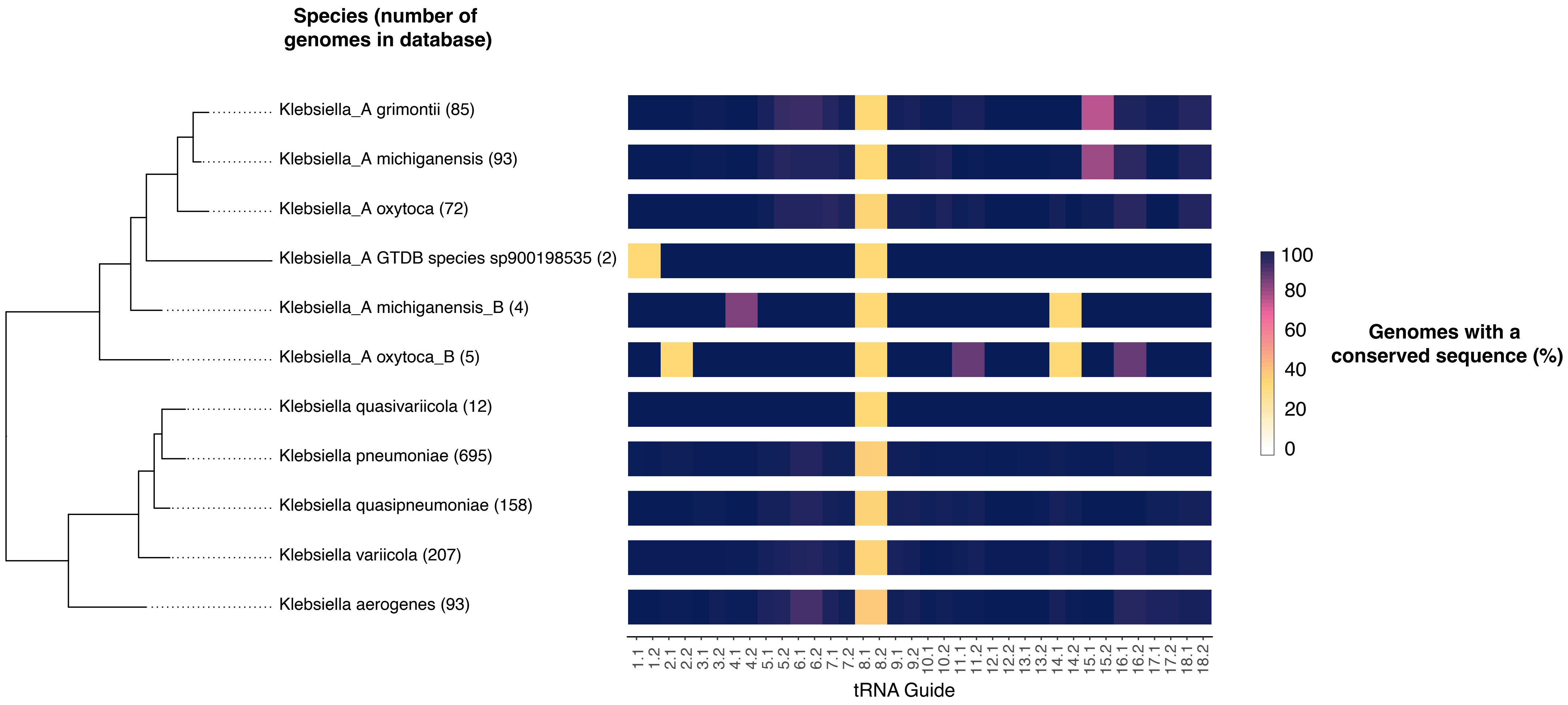

Supplementary Figure 1 - Conservation of tRNA guides across *Klebsiella*

To the left is a neighbour-joining tree of representative genomes from all genera in GTDB r95 classified as *Klebsiella* or *Klebsiella_A* (one genome per species, n=11 species) (Parks et al. 2018; Lee S. Katz 2019). The colour spectrum of the heatmap shows the proportion of genomes matched to guide sequences in a dereplicated version of the full GTDB database for each species (n=1,426 total *Klebsiella* or *Klebsiella_A* genomes, genome count for each species shown in brackets).

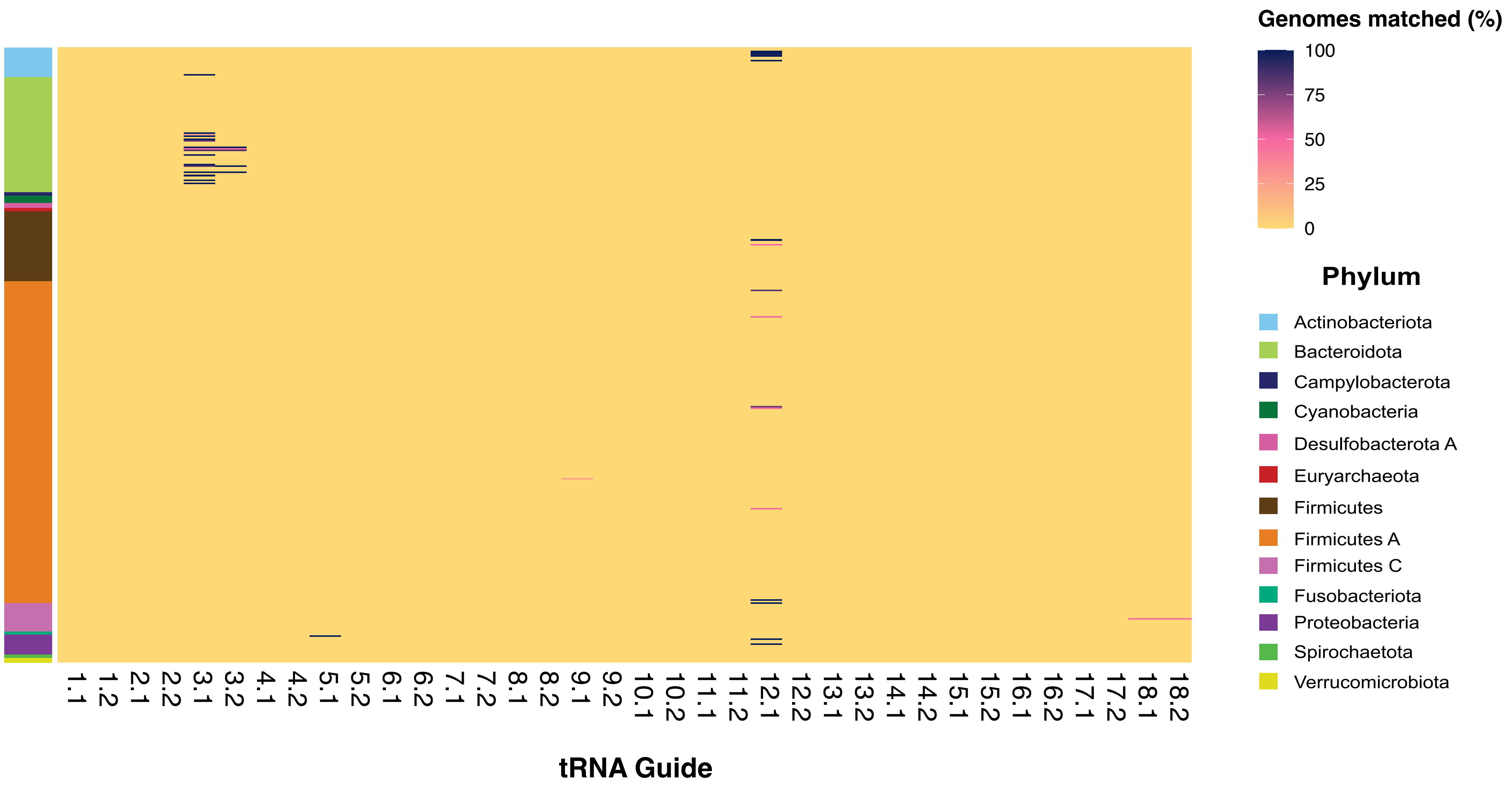

Supplementary Figure 2 - Conservation of tRNA guides amongst commonly observed species outside of *Enterobacterales*
Rows show each of the top 391 most abundant GTDB-defined species present in the human gut (Parks et al. 2018; Almeida et al. 2020). The colour spectrum refers to the proportion of genomes assigned to a given species in the GTDB database that matched with guides. The colour bar to the left of heatmap shows GTDB-defined phylum of each species.

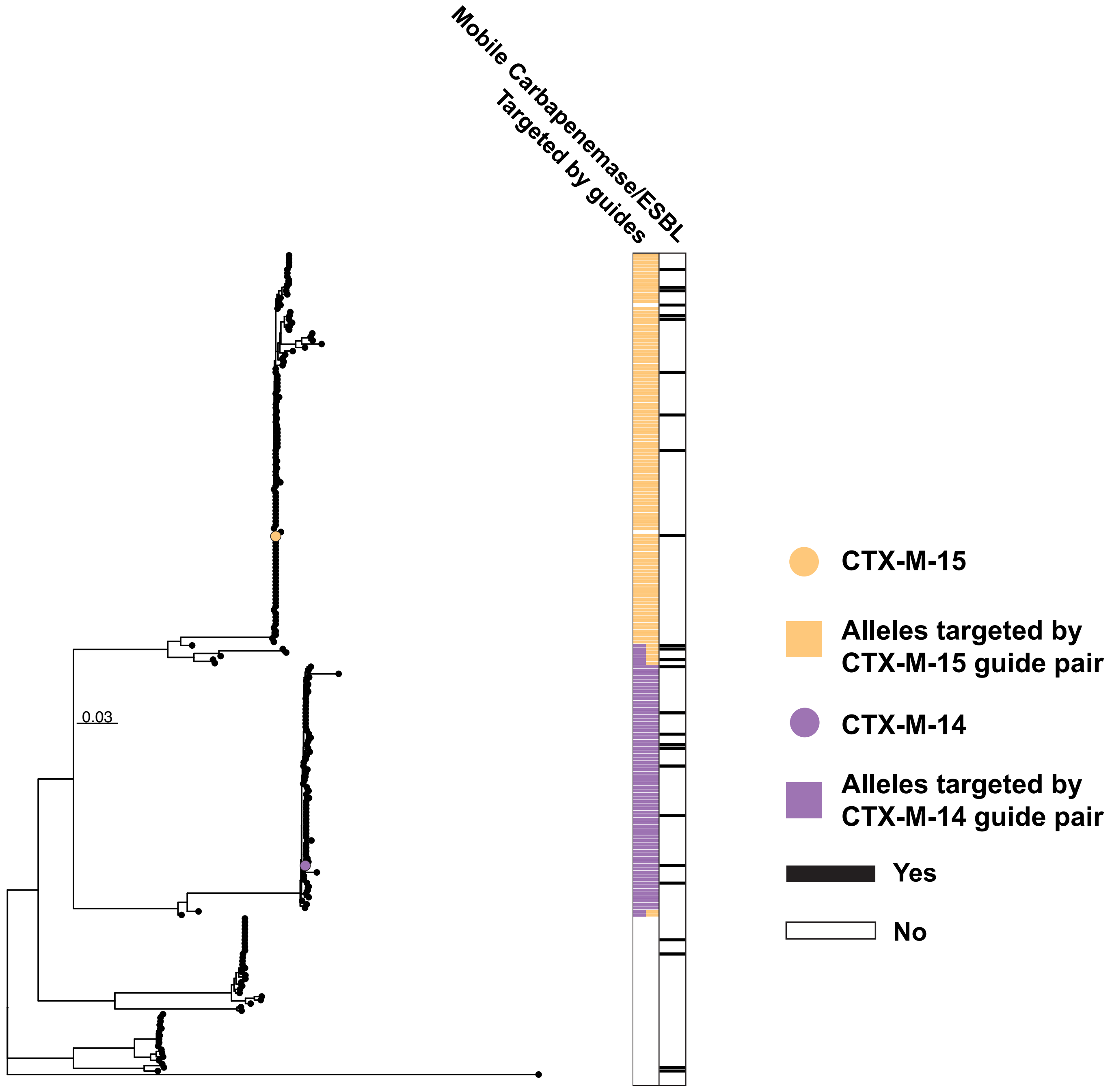

Supplementary Figure 3 - Phylogenetic tree of all *bla*$_{\text{CTX-M}}$ alleles and those targeted by *bla*$_{\text{CTX-M}}$ guides
Generated using BIONJ, rooted at centre (Gascuel 1997; Alcock et al. 2020; Lam et al. 2021). Each dot (node) represent a *bla*$_{\text{CTX-M}}$ allele. The nodes of primary target alleles are coloured, with each allele targeted by the two guide pairs shown as colours in the heatmap. Some alleles are targeted by both pairs- these are represented by bars with both blue and red segments. Alleles that are ESBL/carbapenemase and found in multiple *Enterobacterales* species are also denoted in column two of the heatmap. The scale bar shows the distance correlating to 2% nucleotide variation between clades.

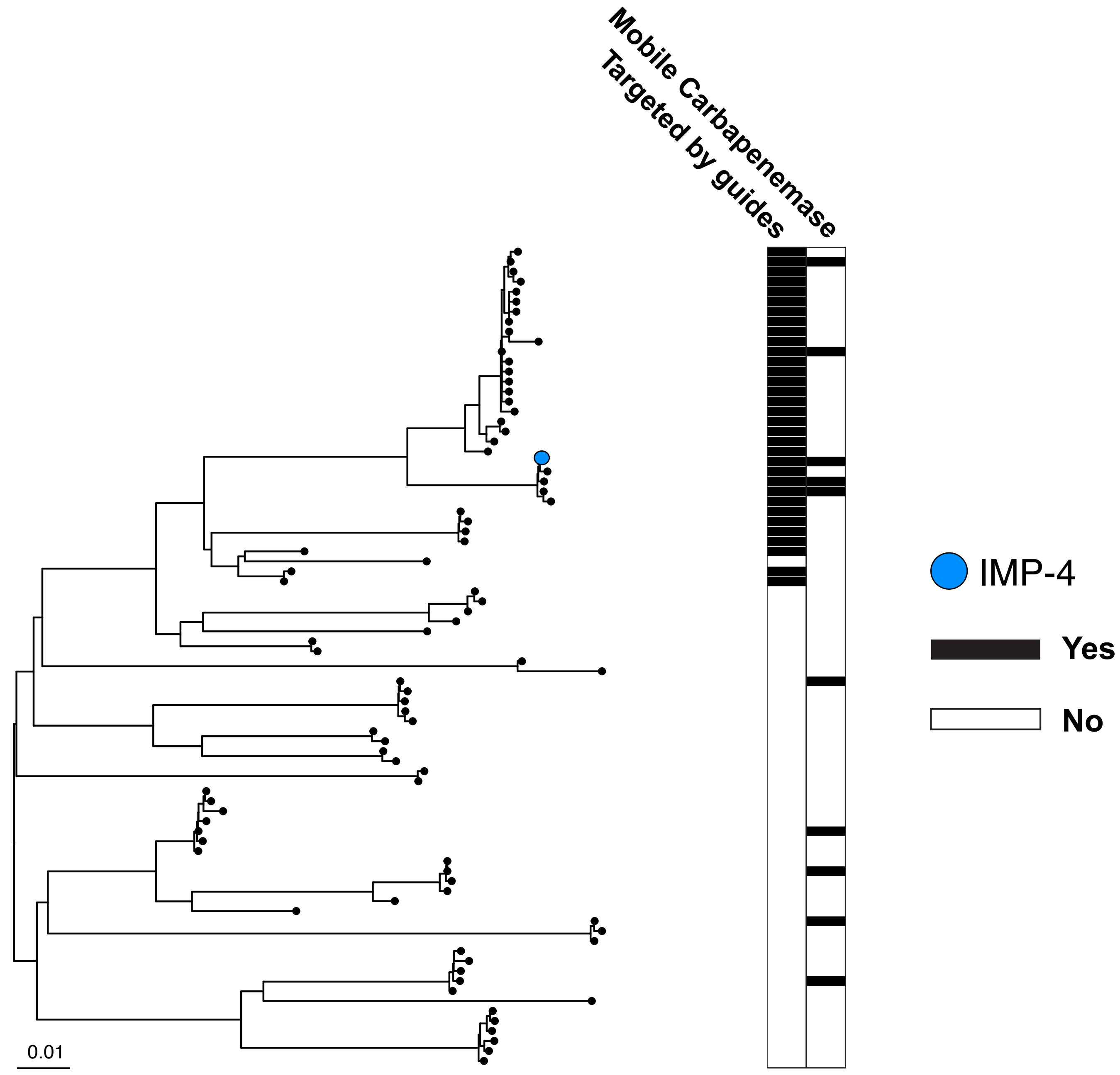

Supplementary Figure 4 - Phylogenetic tree of all *bla*$_{IMP}$ alleles and those targeted by *bla*$_{IMP}$ guides
Generated using BIONJ, rooted at centre (Gascuel 1997; Alcock et al. 2020; Lam et al. 2021). Each dot (node) represent a *bla*$_{IMP}$ allele. The node of the primary target allele is coloured in blue, with each allele targeted by the guide pair shown as black in first row of the heatmap. Alleles that are carbapenemase found in multiple *Enterobacterales* species are also denoted in the second row. The scale bar shows the distance correlating to 1% nucleotide variation between clades.

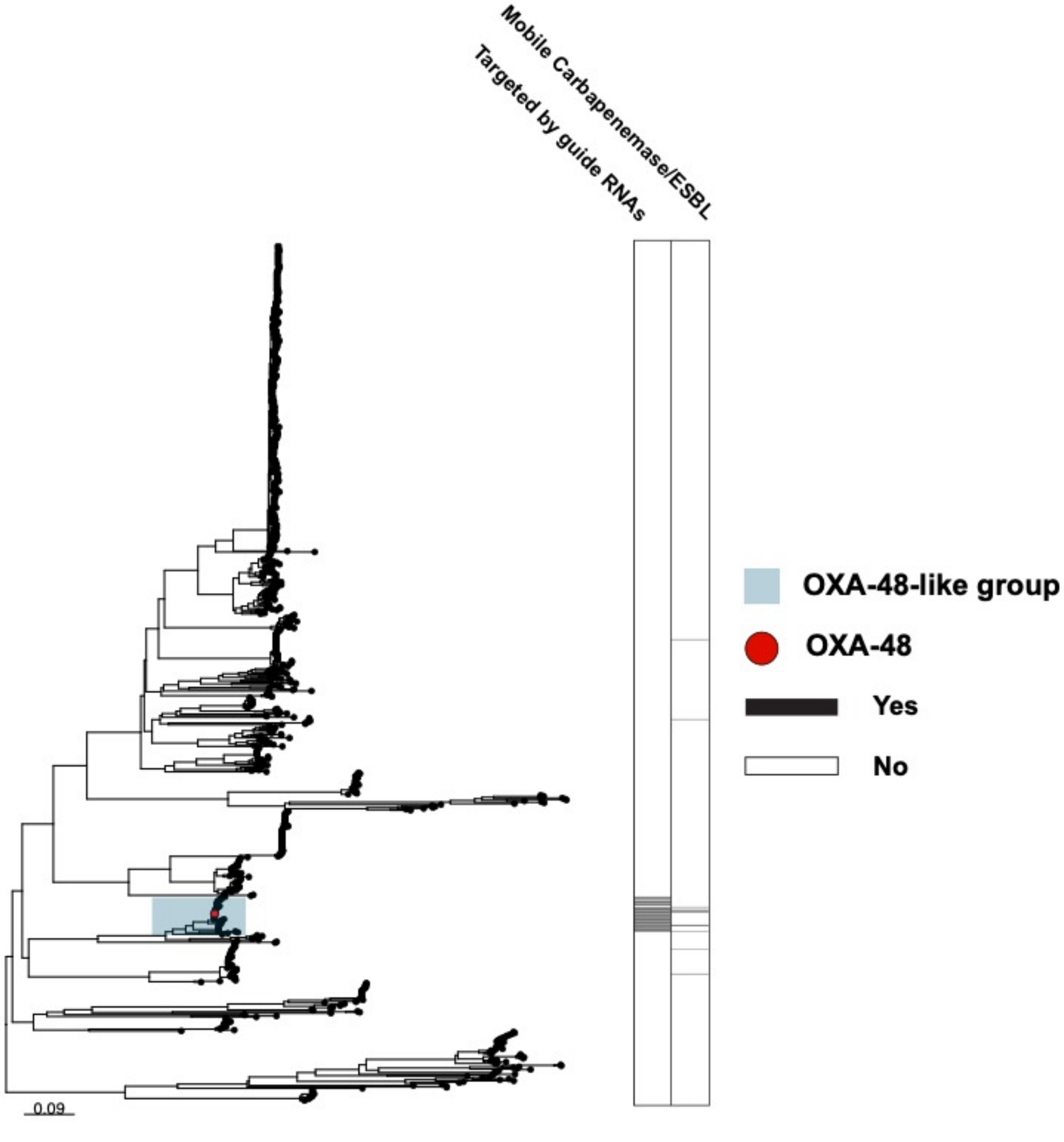

Supplementary Figure 5 Phylogenetic tree of all *bla*$_{OXA}$ alleles and those targeted by *bla*$_{OXA}$ guides

Generated using BIONJ, rooted at centre (Gascuel 1997; Alcock et al. 2020; Lam et al. 2021). Each dot (node) represent a *bla*$_{OXA}$ allele. The node of the primary target allele is coloured in red, with each allele targeted by the guide pair shown as black in the first row of the heatmap. The OXA-48-like group of alleles are highlighted in blue. Alleles that are carbapenemase and found in multiple *Enterobacterales* species are also denoted in the second column. The scale bar shows the distance correlating to 7% nucleotide variation between clades.

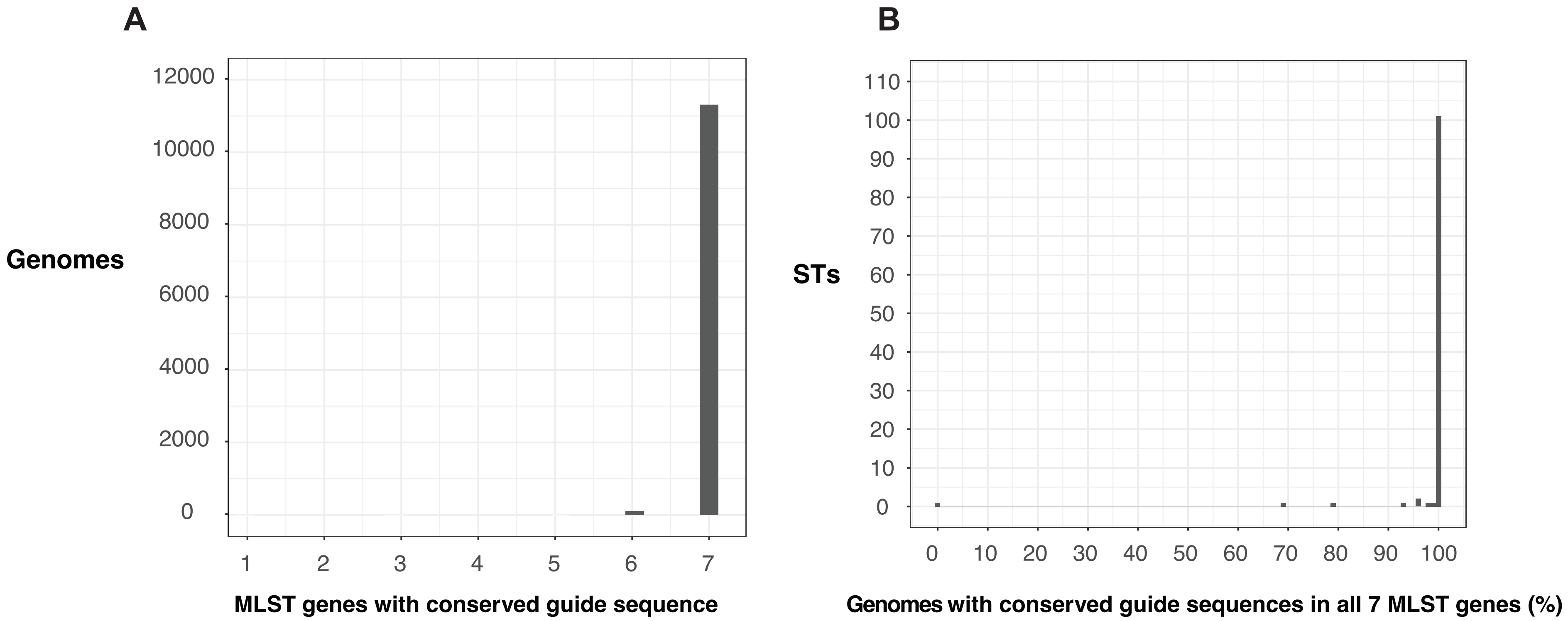

Supplementary Figure 6 – Conservation of MLST-targeting guide pairs in 11,346 *K. pneumoniae* genomes.

Both members of each pair must have a conserved sequence for a match to be reported. **A)** Distribution of the number of MLST genes containing conserved guide sequences for a given genome. **B)** Distribution of number of genomes, for each ST greater than 1% abundance, with a perfect match to all MLST guides.
