# Supplementary material for: Targeted sequencing of Enterobacterales bacteria using CRISPR-Cas9 enrichment and Oxford Nanopore Technologies
Source: mSystems. 2025 Jan 8;10(2):e01413-24. doi: 10.1128/msystems.01413-24 (PMC11834407; doi:10.1128/msystems.01413-24)
Supplement: Figures S8 to S17 — Additional supplemental figures. [file msystems.01413-24-s0005.pdf]

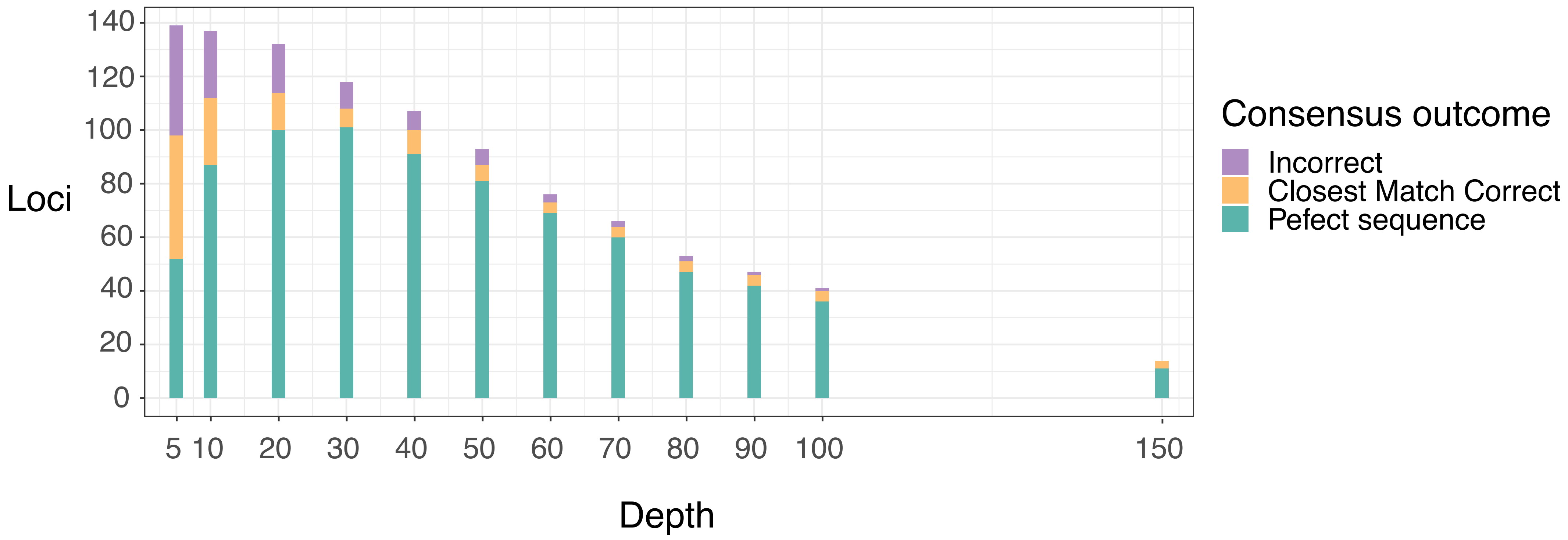

Supplementary Figure 8 – Accuracy of MLST consensus sequences generated from CRISPR-Cas9 enriched ONT data of 20 *K. pneumoniae* isolates.

Depth refers to the number of ontarget reads spanning the MLST-encoding region of a given MLST gene. Consensus outcomes are measured against the completed assembly for that given isolate. 'Incorrect' consensus sequences refer to those with errors in their sequence and with an incorrect closest allele match when aligned against the MLST database. 'Perfect sequence' refers to sequences where the allele is correct, and every base is correct. 'Closest Match Correct' refers to consensus sequences with at least one incorrect base but the top scoring allele is correct when aligned to the MLST database.

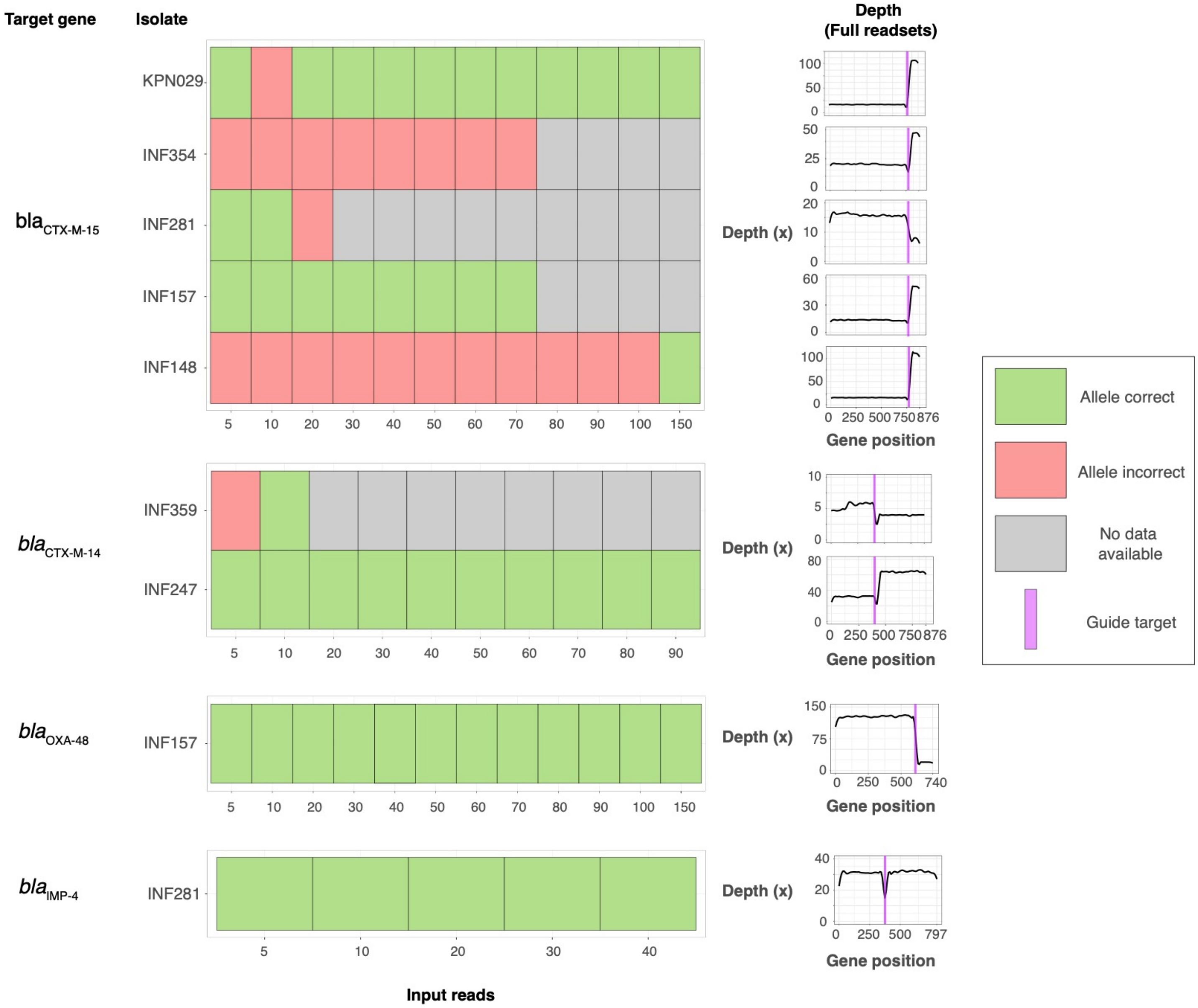

Supplementary Figure 9 – Accuracy of AMR consensus sequences generated from CRISPR-Cas9 enriched ONT data of 20 *K. pneumoniae* isolates.
Input reads represent random subsets of the full collection of reads aligning to the target AMR gene. Consensus outcomes are measured against the completed assembly for that given isolate. 'Incorrect' consensus sequences refer to those with errors in their sequence and with an incorrect closest allele match when aligned against the MLST database. 'Correct' refers to consensus sequences where the top scoring allele is correct when aligned to the CARD AMR database (Alcock et al. 2020).

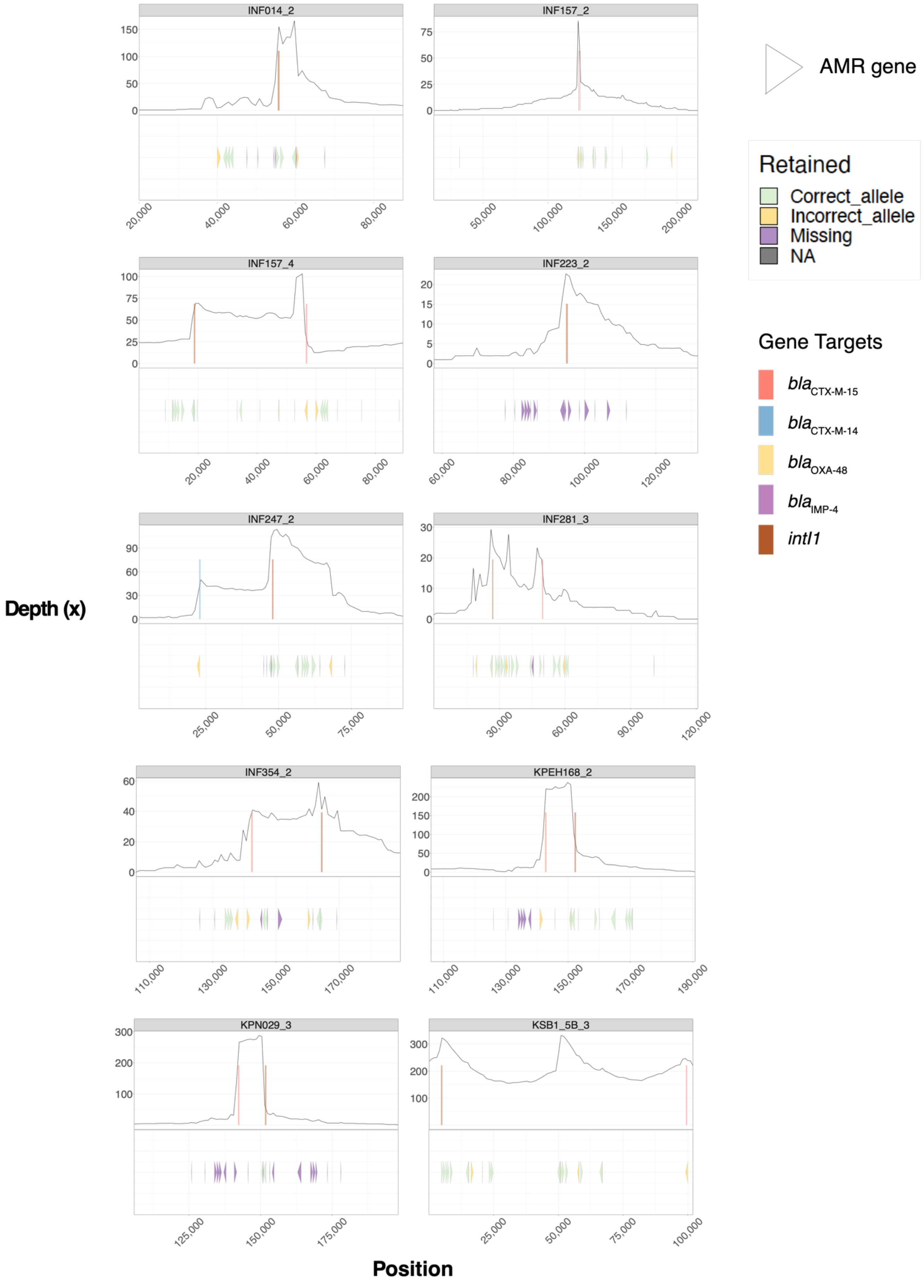

Supplementary Figure 10 – Depth of ontarget reads aligned against *intI1*-containing plasmids across *K. pneumoniae* isolates following CRISPR-Cas9 enrichment and ONT sequencing.
Guide target regions are shown in coloured rectangles. On the bottom panel is the regions of AMR genes on the plasmid, coloured by whether they were absent, retained with the correct allele or retained with an incorrect allele once reads were assembled.

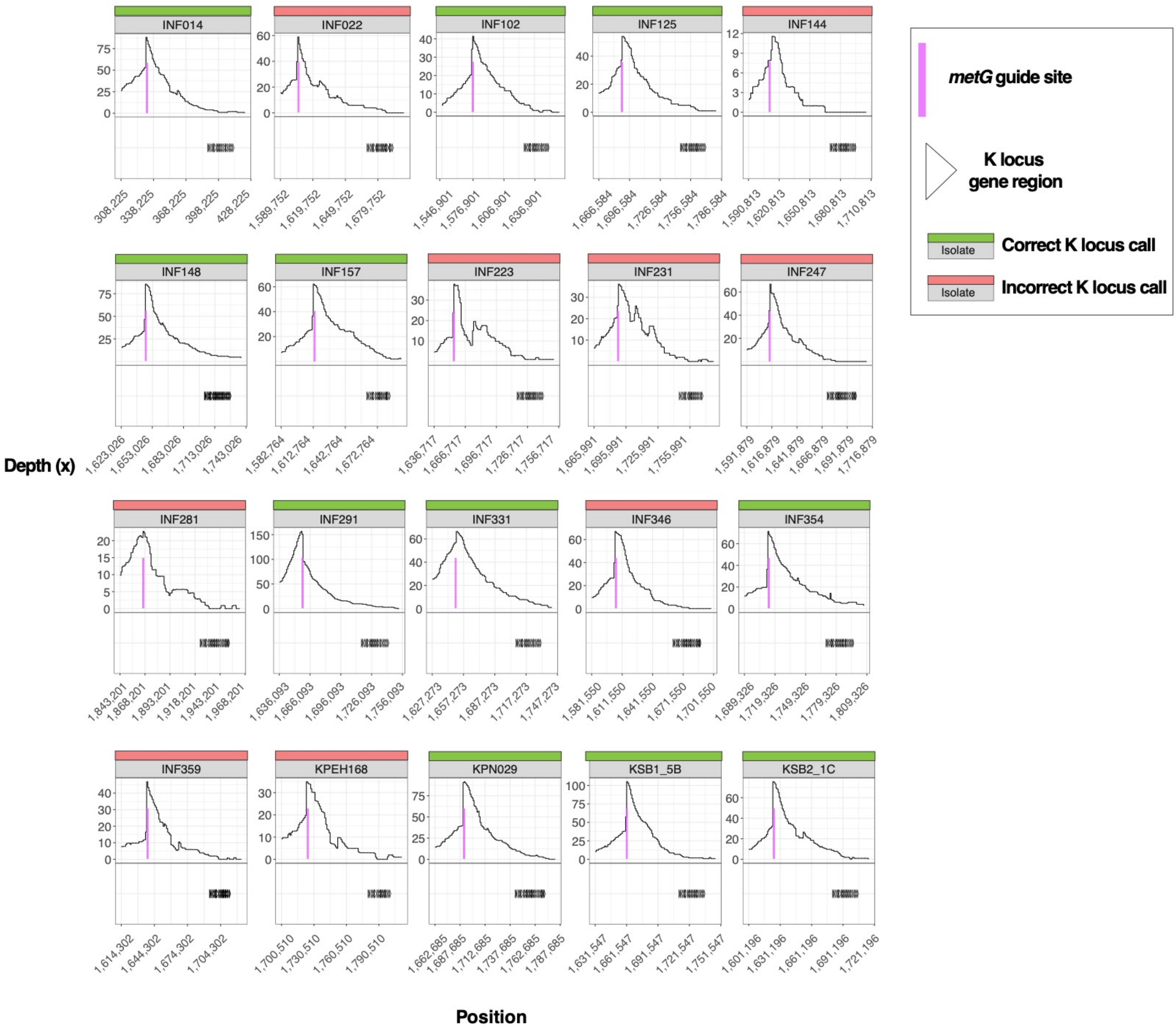

Supplementary Figure 11 – Depth of ontarget reads aligned against the region surrounding the K locus across *K. pneumoniae* isolates following CRISPR-Cas9 enrichment and ONT sequencing. Guide target regions are shown in coloured rectangles. On the bottom panel is the regions of K locus genes.

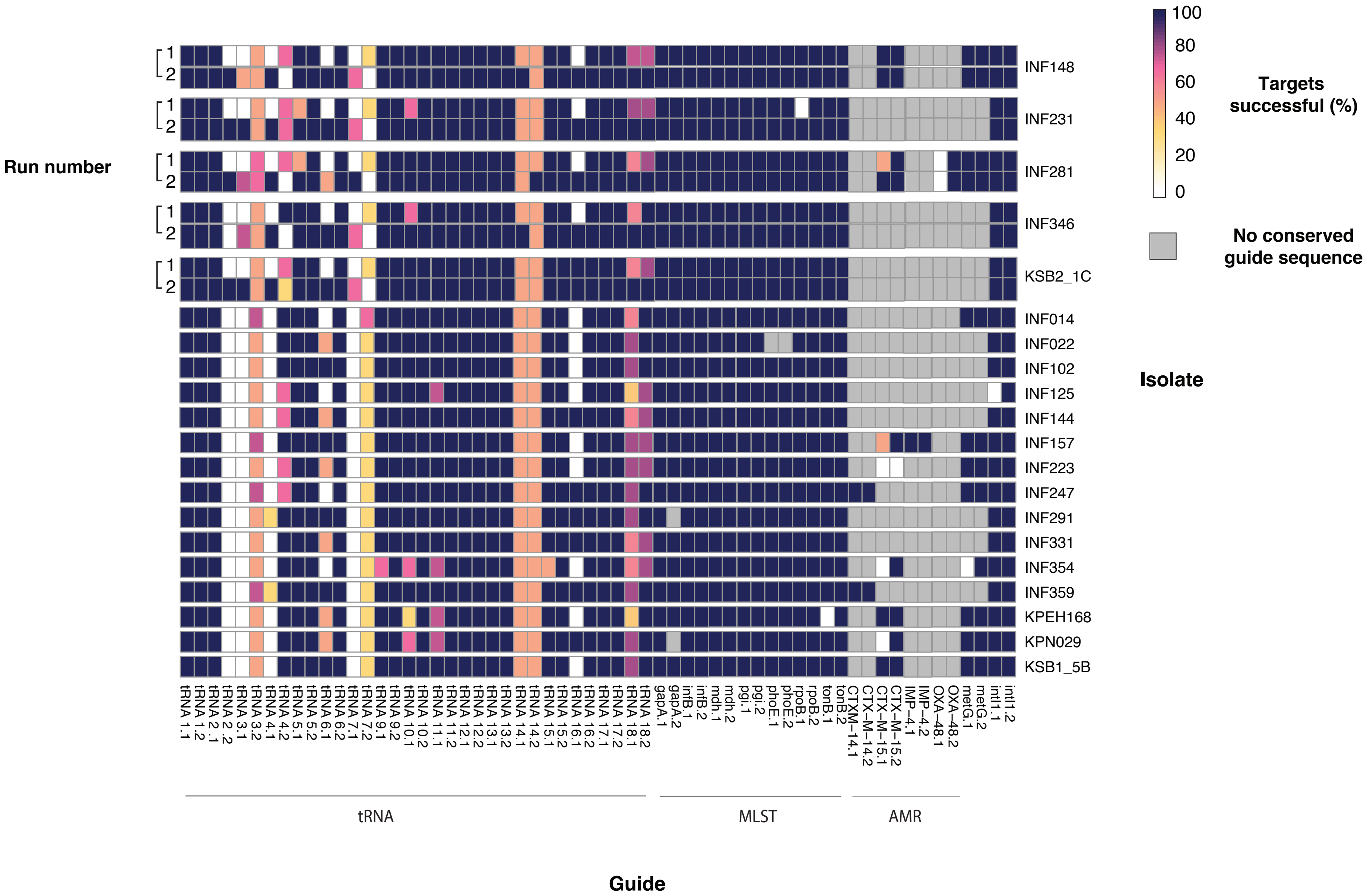

Supplementary Figure 12 – Individual guide performance across CRISPR-Cas9 enriched libraries of 20 *K. pneumoniae* species complex isolates when using one member of each pair at a time. A successful target is defined as when the number of ontarget reads is equal to or greater than 10x median depth of offtarget reads. Run number refers to whether it was the initial experiment (run 1) or the repeat experiment (run 2).

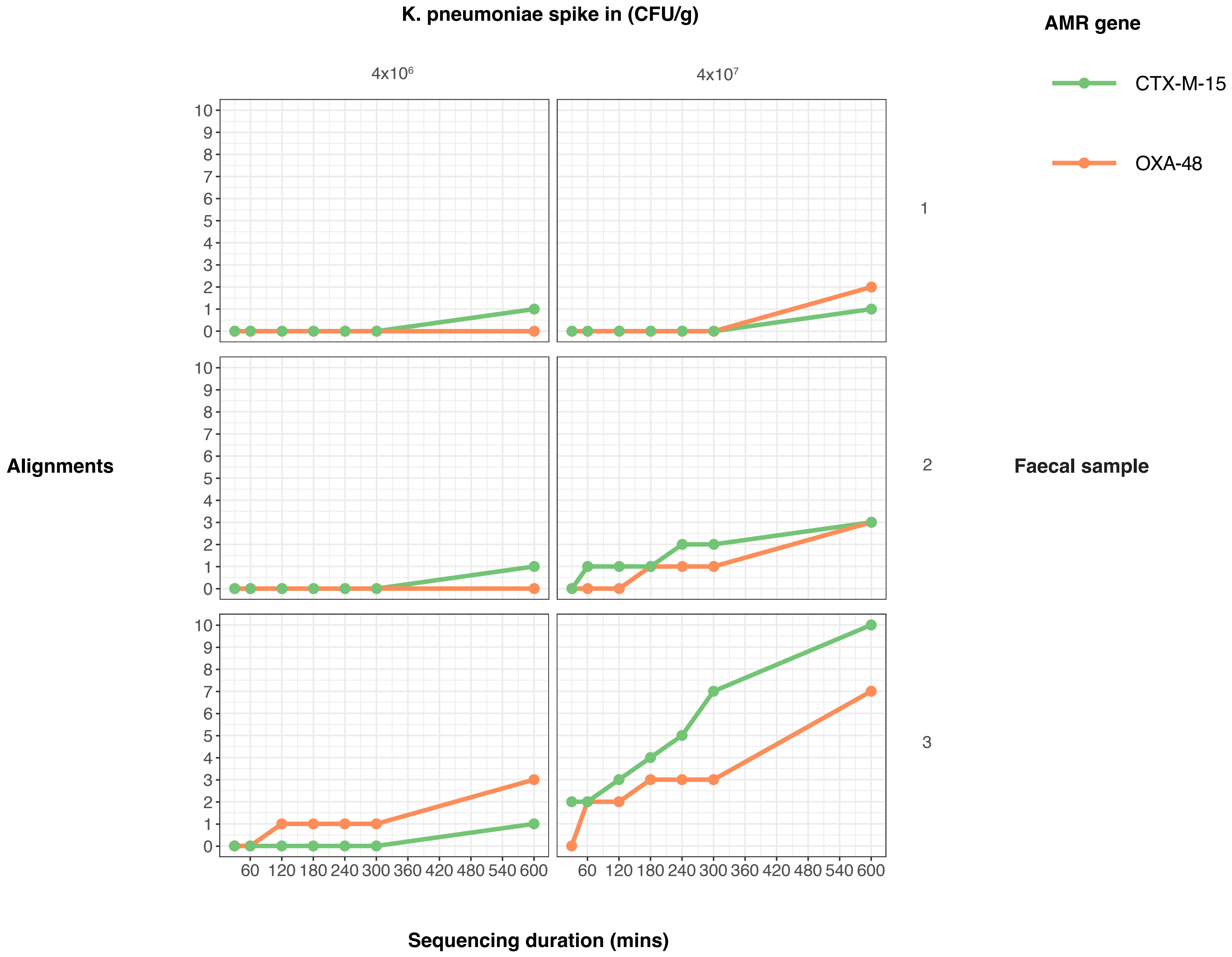

Supplementary Figure 13 – Alignments to target AMR genes in the first ten hours of sequencing following CRISPR-Cas9 enrichment of three human faecal samples spiked with *K. pneumoniae* at 4 x 10^6 – 10^7 CFU/g

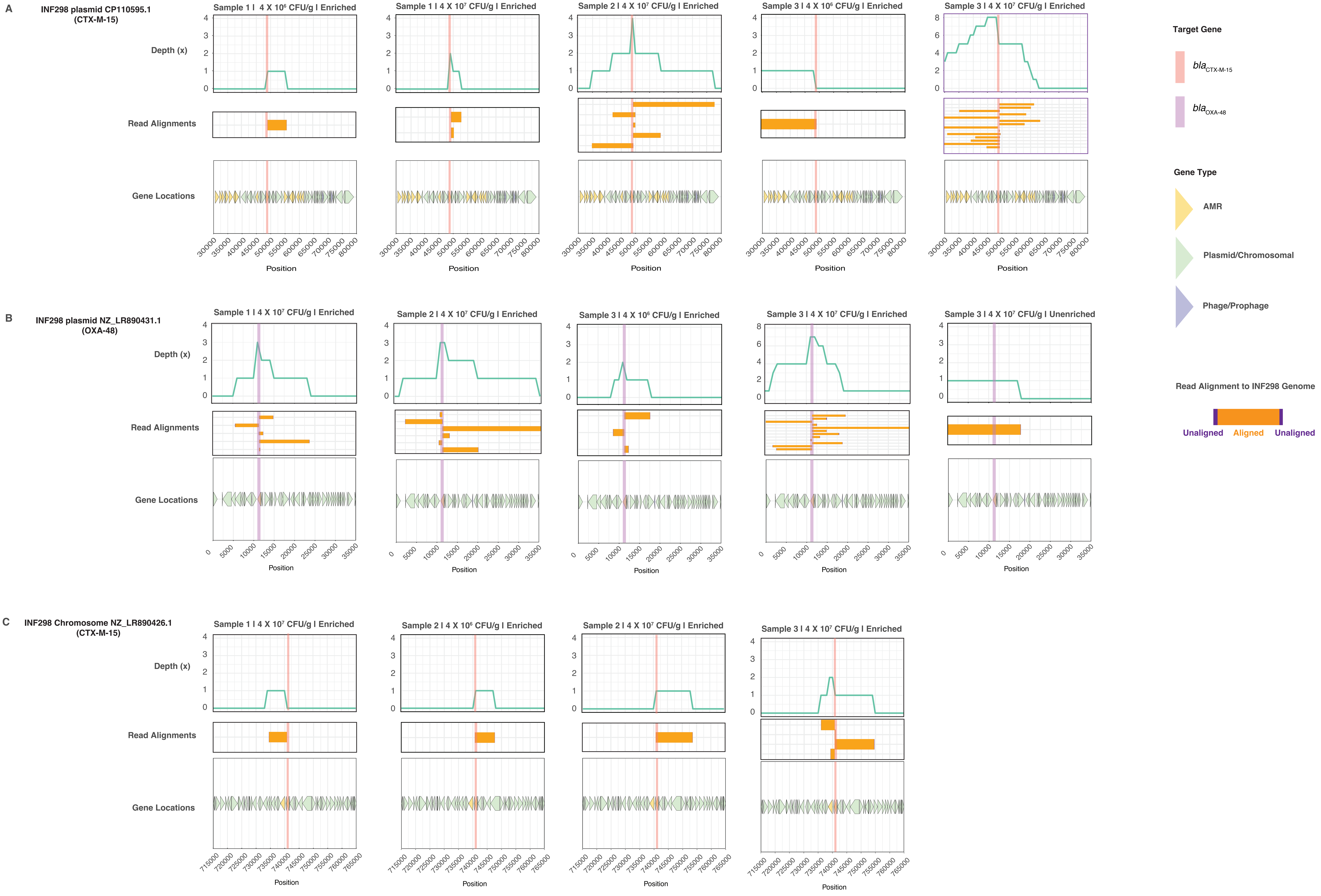

Supplementary Figure 14 - Alignment of AMR reads to spiked INF298 K. pneumoniae genome after enriched and unenriched sequencing of human faecal samples. A) Alignments to the CTX-M-15 gene located on INF298 plasmid CP110595.1 **B)** Alignments to the OXA-48 gene located on INF298 plasmid NZ_LR890431.1 **C)** Alignments to the CTX-M-15 gene located on INF298 chromosome NZ_LR890426.1.

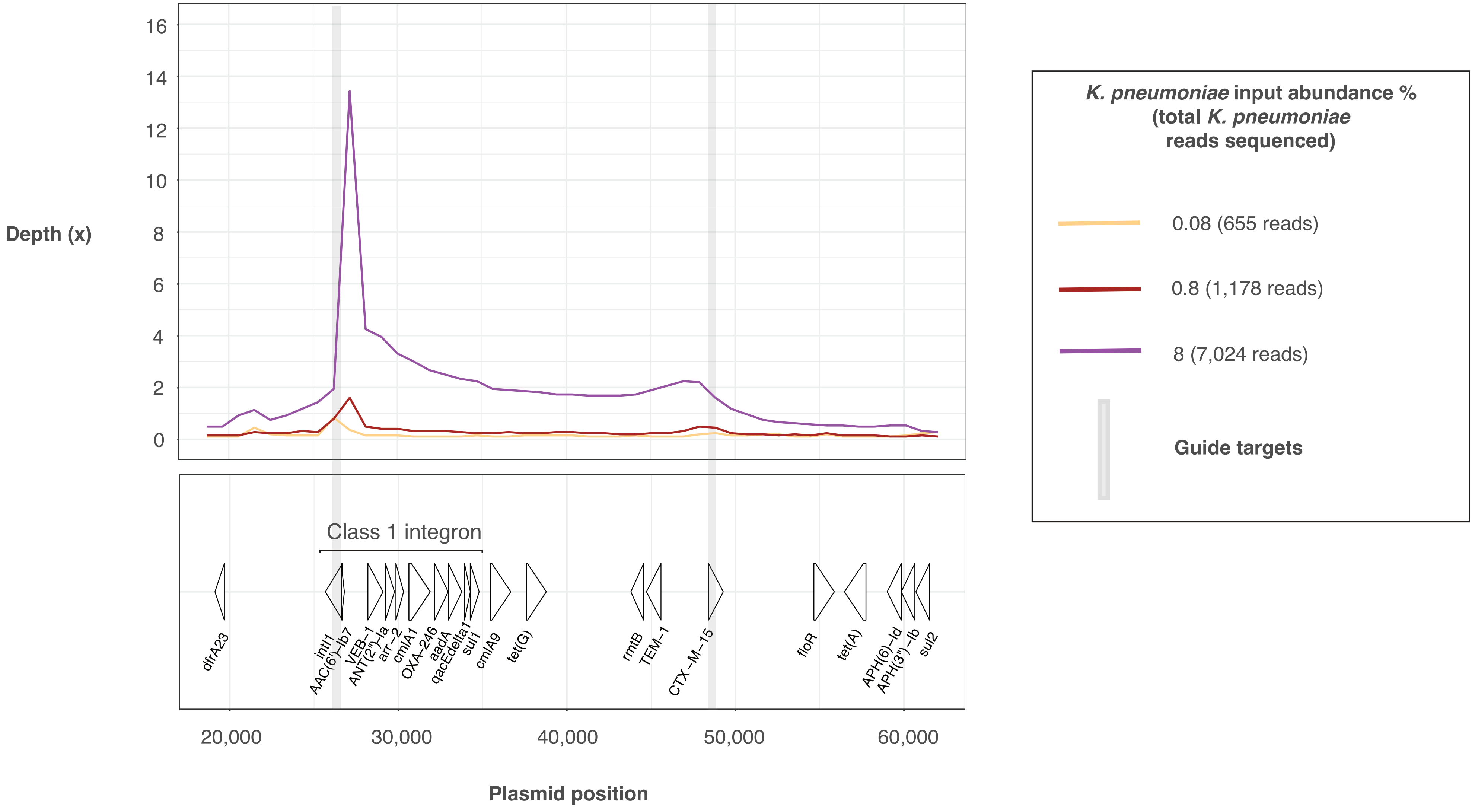

Supplementary Figure 15 – Depth of an enriched plasmid following sequencing of a mock microbial mixture spiked with *K. pneumoniae* strain INF298 DNA at 0.8 – 8% relative abundance.

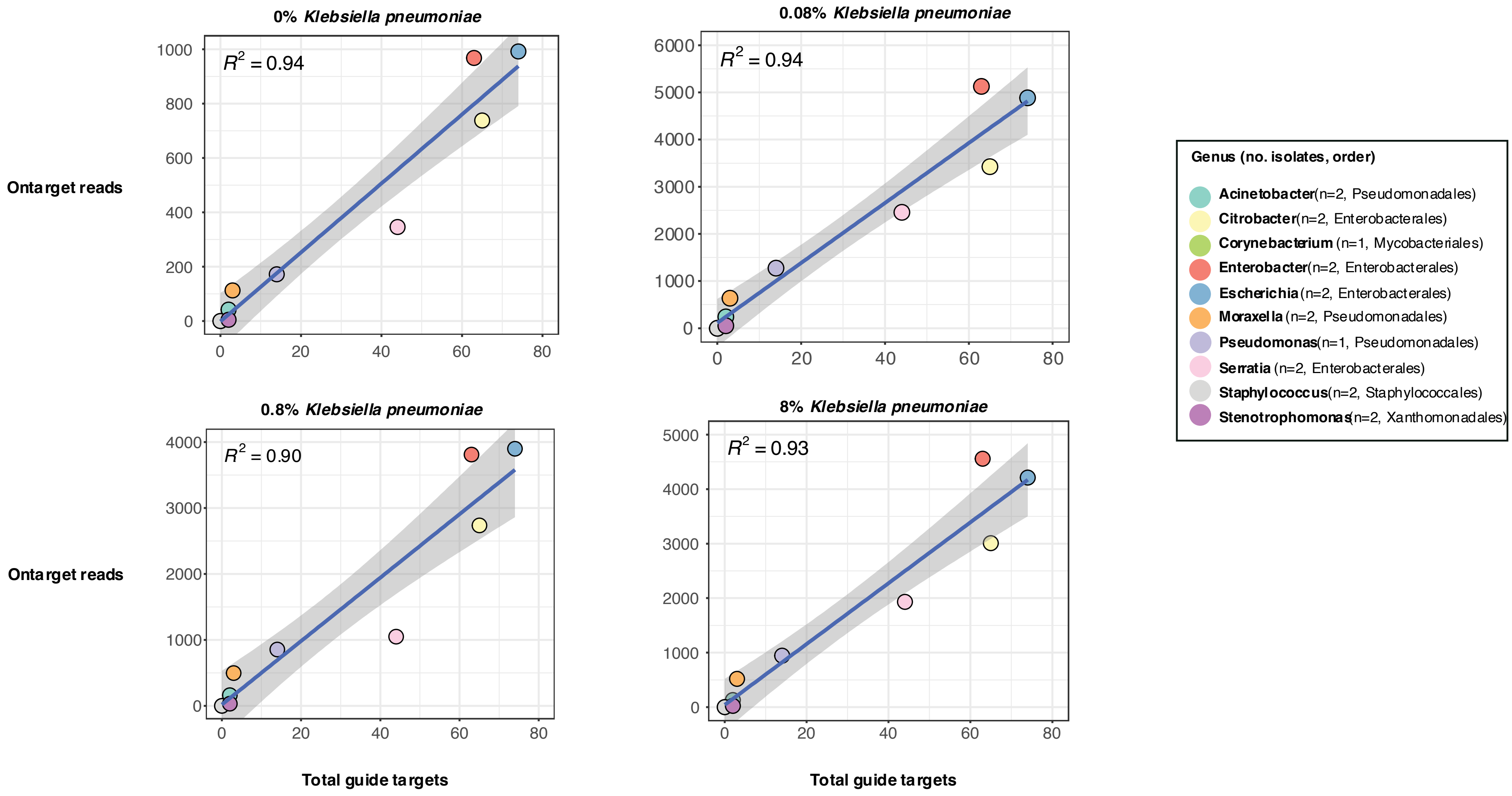

Supplementary Figure 16 – Relationship between guide conservation and enrichment success amongst isolates in a bacterial community mixture. Plots show a linear regression between the total guide targets and total ontarget reads for isolates of a given genus. All isolates were included in the mixture at the same abundance except for *K. pneumoniae*, which was included at abundances labelled on top of each plot. *K. pneumoniae* statistics were excluded from linear regression analyses due to different abundance between libraries.

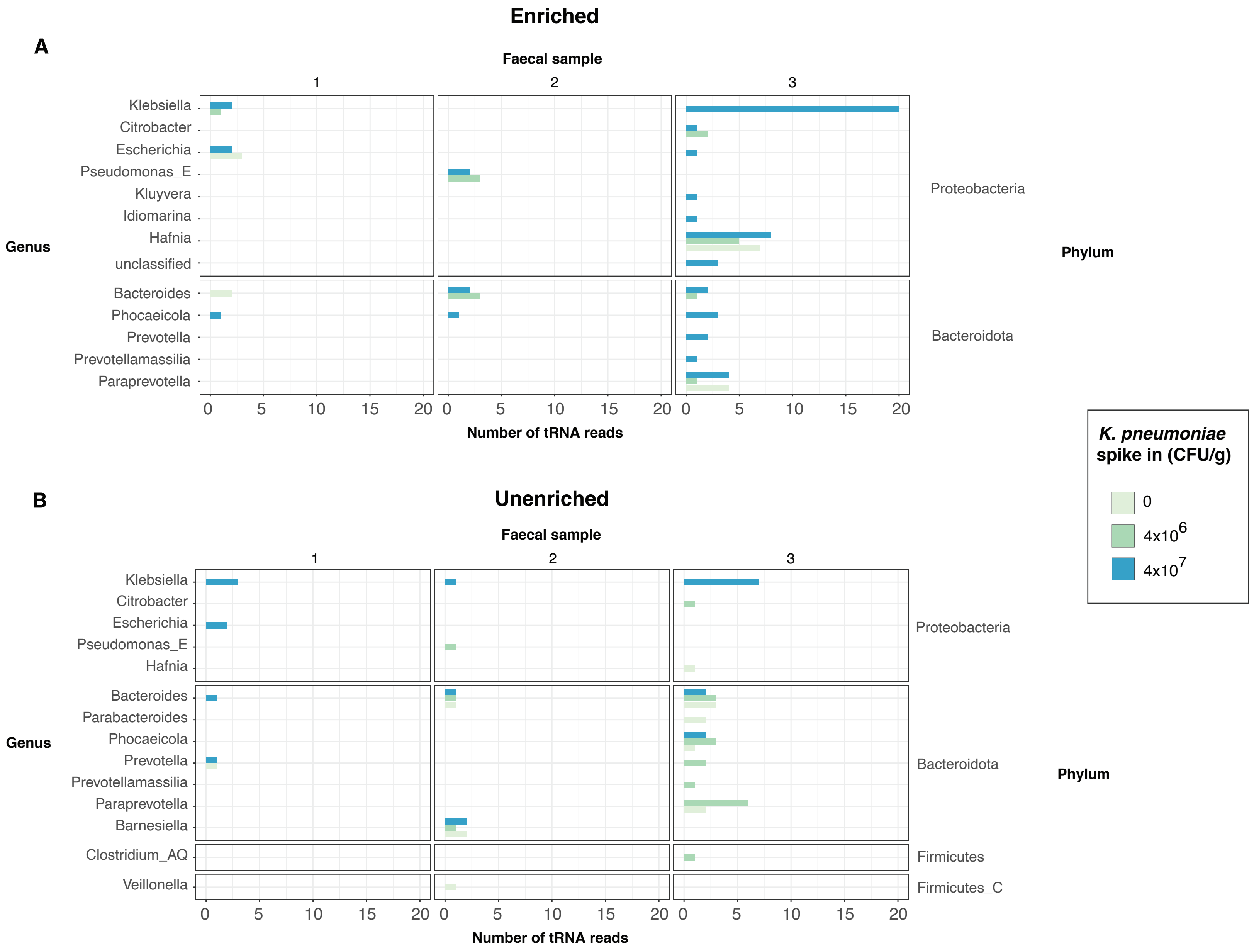

Supplementary Figure 17 – Taxonomic classification of reads aligning to target tRNA genes following sequencing of three human faecal samples (Parks et al. 2018; Wood et al. 2019).

A) Classification of reads aligning to tRNA genes following CRISPR-Cas9 enriched sequencing. **B)** Classification of reads aligning to tRNA genes following unenriched sequencing**.**
