# Supplementary material for: Targeted sequencing of Enterobacterales bacteria using CRISPR-Cas9 enrichment and Oxford Nanopore Technologies
Source: mSystems. 2025 Jan 8;10(2):e01413-24. doi: 10.1128/msystems.01413-24 (PMC11834407; doi:10.1128/msystems.01413-24)
Supplement: Figure S7 — Depth plots of all enriched isolate sequencing. [file msystems.01413-24-s0004.pdf]

Supplementary Figure 7 – Sequencing depth of *Enterobacterales* isolates containing conserved guide sequences following CRISPR-Cas9 enrichment and ONT sequencing.

Contig number is shown under each isolate code.Total depth is shown as the dark blue line, with offtarget depth in light blue. Target gene regions are shown as coloured rectangles.

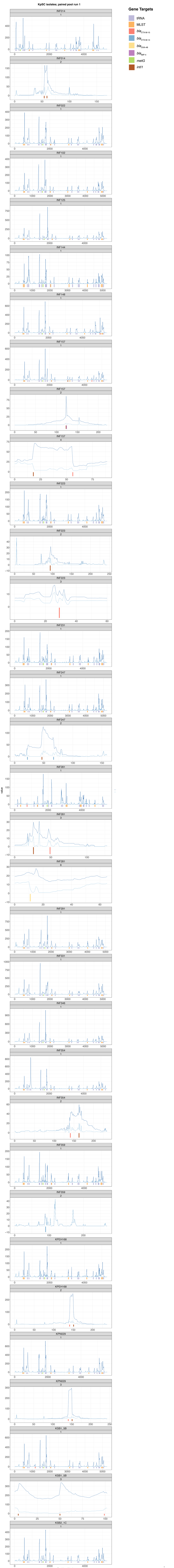

KpSC isolates; paired pool run 2

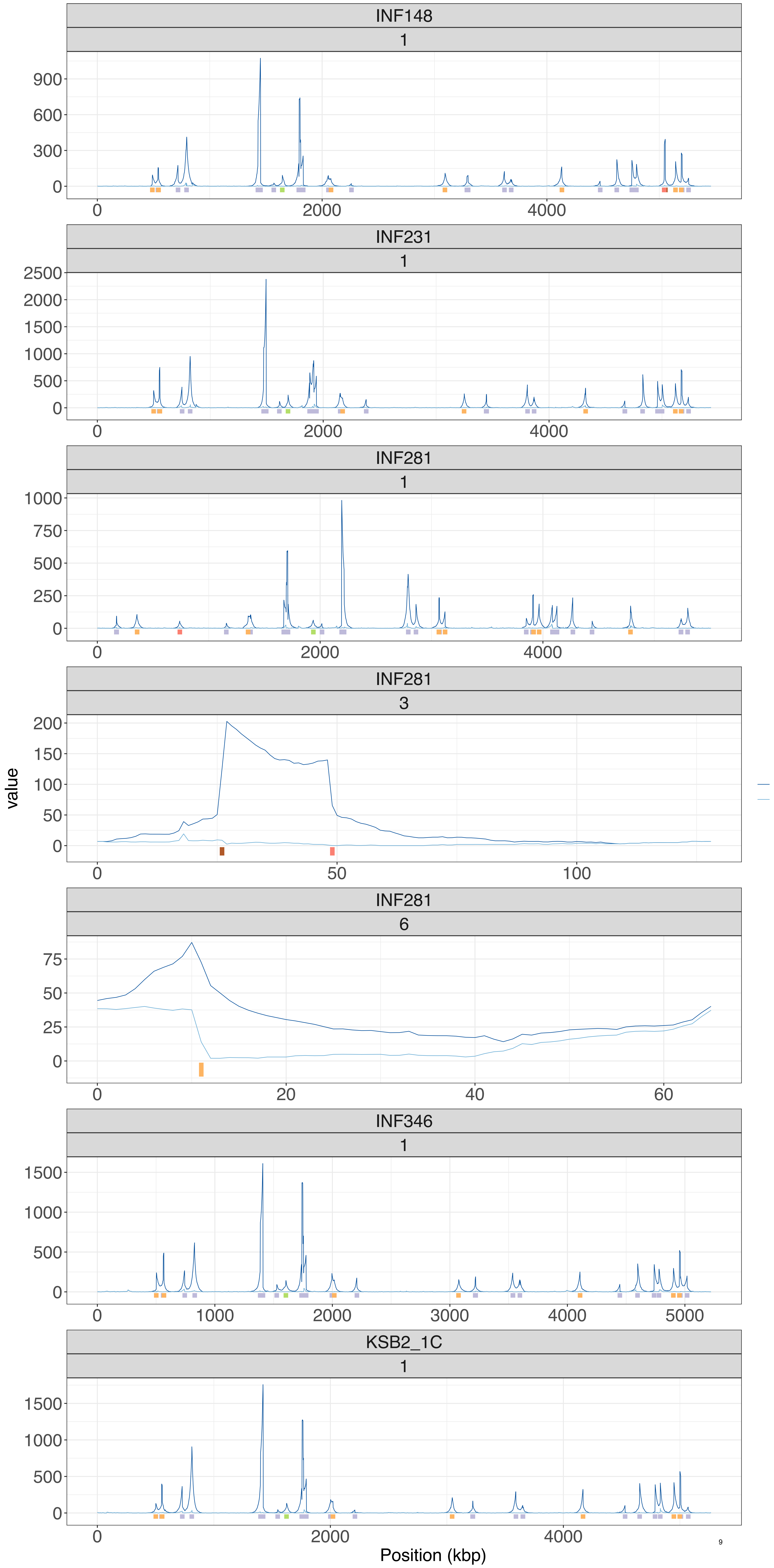

KpSC isolates; unpaired 1 guide pool

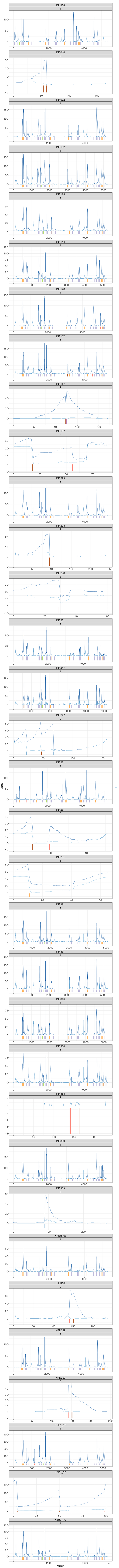

KpSC isolates; unpaired 2 guide pool

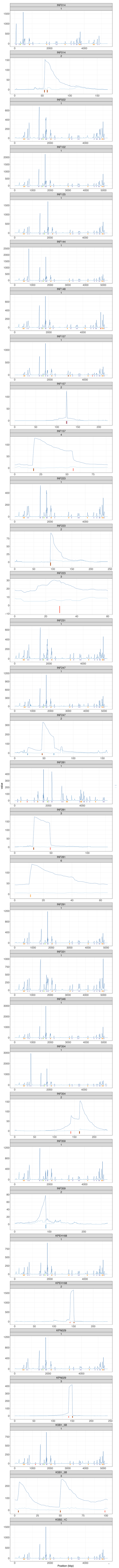

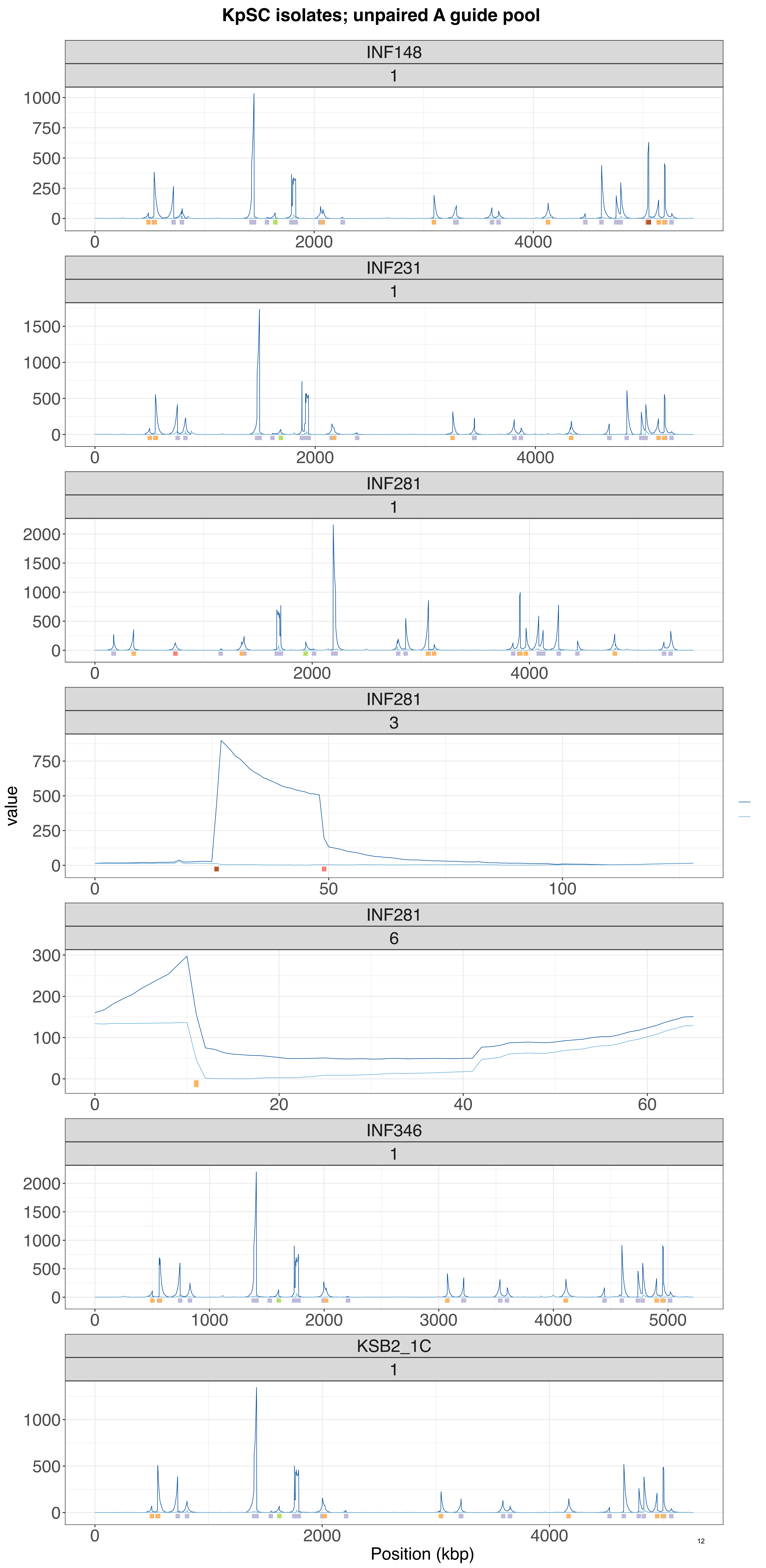
KpSC isolates; unpaired A guide pool
INF148
1
INF231
1
INF281
1
INF281
3
INF281
6
INF346
1
KSB2_1C
1
value
Position (kbp)
12

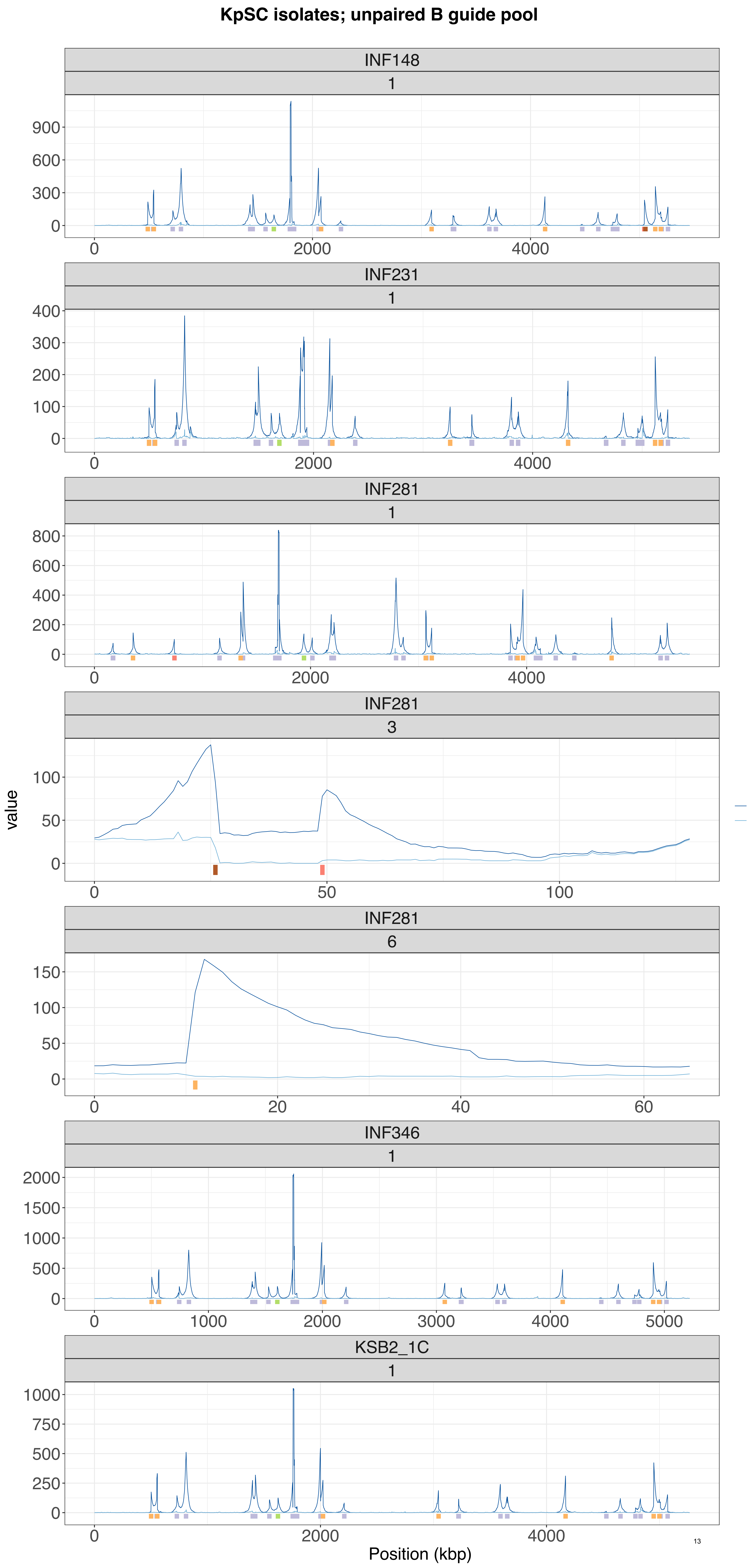
KpSC isolates; unpaired B guide pool
INF148
1
INF231
1
INF281
1
INF281
3
INF281
6
INF346
1
KSB2_1C
1
value
Position (kbp)

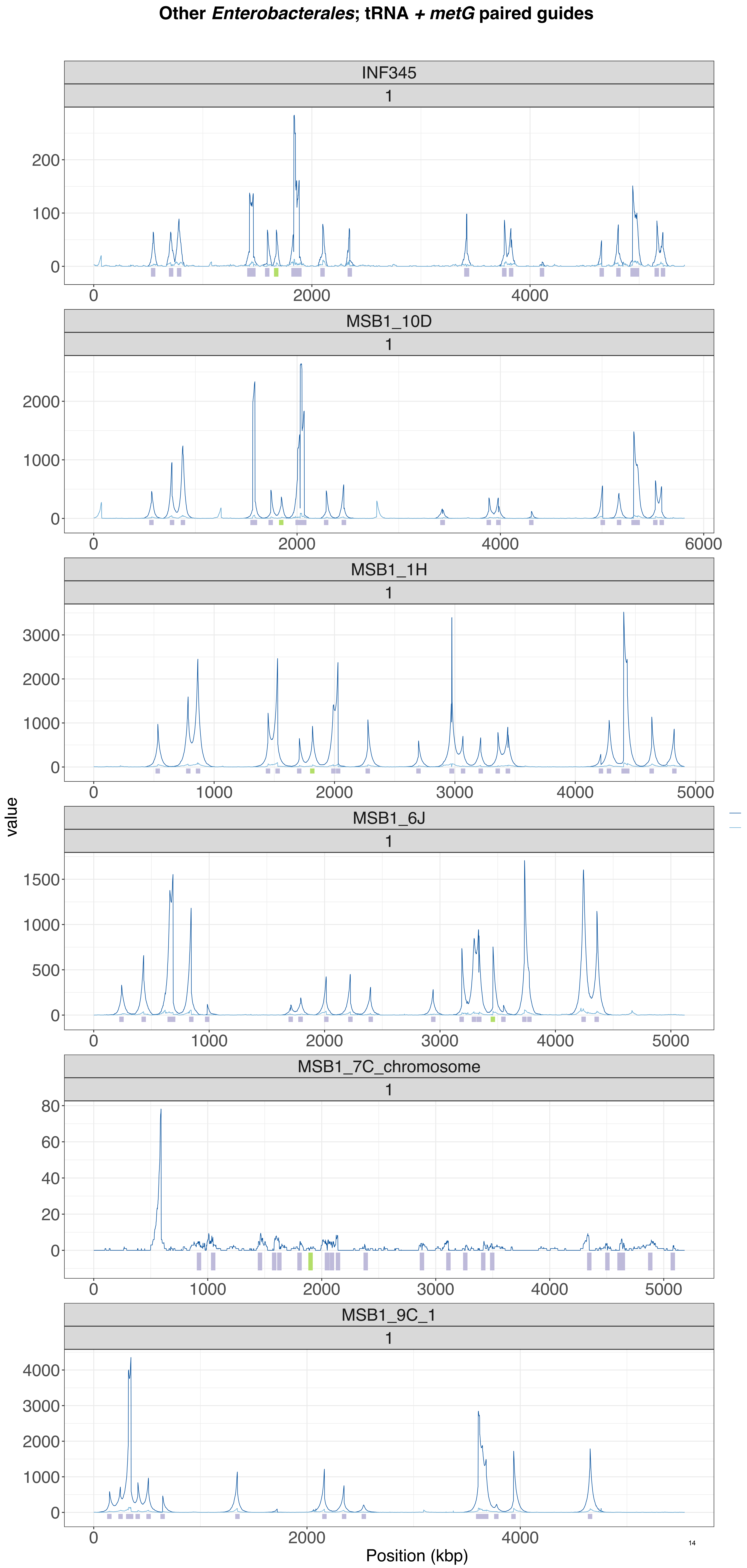
Other Enterobacterales; tRNA + metG paired guides
INF345
1
MSB1_10D
1
MSB1_1H
1
MSB1_6J
1
MSB1_7C_chromosome
1
MSB1_9C_1
1
value
Position (kbp)
